# What Do Asexual Women Want? A Propensity Score Matching Study of Preferred Relationship Options and Ideal Partner Preferences

**DOI:** 10.1007/s10508-025-03365-2

**Published:** 2026-02-05

**Authors:** Paula C. Bange, Laura J. Botzet, Amanda A. Shea, Virginia J. Vitzthum, Tanja M. Gerlach

**Affiliations:** 1 Department of Developmental Psychology, Tilburg University, Simon Building S314, PO Box 90153, 5000 LE Tilburg, The Netherlands; 2 Biological Personality Psychology, University of Goettingen, Goettingen, Germany; 3 Leibniz ScienceCampus Primate Cognition, Goettingen, Germany; 4 Clue By BioWink GmbH, Berlin, Germany; 5 CEMCOR, Department of Medicine, University of British Columbia, Vancouver, BC Canada; 6 Department of Anthropology, Indiana University, Bloomington, IN USA; 7 Leibniz Institute for Educational Trajectories (LIfBi), Bamberg, Germany; 8 School of Psychology, Queen’s University Belfast, Belfast, UK

**Keywords:** Asexuality, Propensity score matching, Preferred relationship options, Ideal partner preferences, Personality

## Abstract

Research on whether asexual individuals desire (romantic) relationships and, if so, how they picture their ideal relationship has been growing in the past few years. However, less is known about the preferred attributes of an ideal partner in such relationships and whether these partner(ship) preferences are different from what heterosexual individuals want. The goal of the present study was to compare the types of preferred relationships and the ideal characteristics of a long-term partner of self-identified asexual and heterosexual women. Additionally, we examined differences in characteristics of asexual and heterosexual women using self-evaluations of the same attributes used for the partner preference ratings. We used data from the Ideal Partner Survey, a large-scale, multinational online study. Of 51,775 participants, 51,328 identified as heterosexual (*M*_*age*_ = 25.13 years) and 447 identified as asexual (*M*_*age*_ = 24.03 years). To create comparable samples for analyses, each asexual person was matched with a heterosexual person using propensity score matching (relationship options sample = 646, partner preference sample = 780, self-rating sample = 772). Compared to heterosexual women, asexual women were less interested in purely sexual relationships and more interested in emotionally romantic and alternative types of committed relationships as well as not being in any relationship (“single”). Asexual women placed less importance on all partner preference attributes, except educated and intelligent. They also consistently rated themselves lower on all attributes than heterosexual women. These findings suggest distinct differences between asexual and heterosexual women in their relationship interests, partner preferences, and self-perceived characteristics.

**Supplementary Information:**

The online version contains supplementary material available at 10.1007/s10508-025-03365-2.

## Introduction

The nearly universal assumption that all individuals have or will experience sexual desire is mistaken. Asexuality has long been ignored or even pathologized (Hille et al., 2023). However, in recent years, its public visibility has been increasing, demonstrated by asexual characters appearing on mainstream television shows (e.g., *Sex Education*), its recognition as a sexual orientation (e.g., Bogaert, 2015), and a growing body of research on asexuality. Sexual attraction has been suggested as a mechanism that guides partner preferences (Scheller et al., 2023). Thus, in the absence of or with reduced sexual attraction, relationship interests and partner preferences may differ. The goal of the current study was to examine differences between asexual and heterosexual individuals in how they envision their ideal relationship. In these relationships, are there differences in how asexual and heterosexual individuals picture an ideal partner? And do they differ in what picture they have of themselves? By comparing the preferences of asexual and heterosexual individuals, we can better understand the role that sexual attraction may play in relationship and partner selection processes.

### Asexuality

Asexuality is usually defined as having little or no sexual attraction to others (e.g., Bogaert, 2004; Greaves et al., 2021) and is experienced by approximately 0.4 to 1% of the population (Aicken et al., 2013; Bogaert, 2004; Greaves et al., 2017). Asexuality has been conceptualized as a sexual orientation like homosexuality, heterosexuality, and bisexuality (e.g., Bogaert et al., 2018). Of note, asexuality includes a spectrum of sub-identities such as graysexual (individuals who fall somewhere in-between asexual and sexual) and demisexual (individuals who experience sexual attraction only after forming a deep emotional bond; Carrigan, 2011; Copulsky & Hammack, 2023).

Following Diamond’s (2003) biobehavioral model of love and sexual desire, sexual attraction and romantic attraction are distinct and function independently of one another, serving different evolutionary purposes, reproduction and pair bonding, respectively. Hence, while asexual individuals may experience little to no sexual attraction, they may experience romantic attraction and wish to be in emotionally intimate relationships. Conversely, they may also identify as aromantic, meaning that they do not experience romantic attraction either (Copulsky & Hammack, 2023). Aromanticism is relatively common among asexual individuals, with Antonsen et al. (2020) reporting that 26% and Clark and Zimmerman (2022) reporting that 37% identify as aromantic. Allosexuality refers to individuals who experience sexual attraction. Compared to allosexual individuals, asexual individuals are more likely to be women or gender diverse (Antonsen et al., 2020; Bogaert, 2004; Greaves et al., 2017), are less likely to be in a romantic relationship (Aicken et al., 2013; Bogaert, 2004; Greaves et al., 2017), have fewer lifetime sexual partners and later onset of sexual activity (if at all) engage in sexual activities less frequently (Bogaert, 2004), and are more likely to report never having had sexual intercourse (Aicken et al., 2013).

### Empirical Evidence

#### Preferred Relationship Options

Research on the intimate lives of asexual individuals uncovered substantial heterogeneity in whether asexual individuals seek relationships and how much and what kind of intimacies they desire in these relationships (Brotto et al., 2010; Carrigan, 2011; Dawson et al., 2016; Higginbottom, 2024; Scherrer, 2008; Van Houdenhove et al., 2015).

Some asexual individuals neither want sexual nor romantic partnerships (Brotto et al., 2010; Carvalho & Rodrigues, 2024; Higginbottom, 2024; Scherrer, 2008). While for some this may arise from being aromantic (Carvalho & Rodrigues, 2022; Tessler, 2023), others may be avoiding the difficulties that come with finding a partner and/or maintaining a relationship. Being single may offer a safe alternative to getting hurt in the process of finding someone that is either also asexual or accepts one’s asexuality (Higginbottom, 2024). Negotiating and compromising on all forms of physical intimacy may also be stressful and therefore avoided by some (Dawson et al., 2016; Higginbottom, 2024; Van Houdenhove et al., 2015). Thus, asexual individuals are likely to endorse being non-partnered more so than other sexualities.

However, many asexual individuals do want relationships, especially the closeness and intimacy that comes with them. In previous studies their ideal relationship was often described as not involving sexual intercourse (Dawson et al., 2016; Hille et al., 2020). How much intimacy is wanted varies greatly among asexual individuals (Dawson et al., 2016; Higginbottom, 2024; Hille, 2023; Scherrer, 2008) and seems to be related to the individual attitudes towards sex (Carrigan, 2011) and their romantic orientation (Carvalho & Rodrigues, 2022). Reasons for asexual individuals to engage in sexual activities are diverse: Curiosity, feelings of societal pressure to have sex, or doing it for the sake of the relationship, as a favor for the partner (Dawson et al., 2016; Higginbottom, 2024; Hille et al., 2020; Van Houdenhove et al., 2015).

While some asexual individuals seek monogamous relationships (Scherrer, 2008, 2010), others are open to non-monogamous relationships (Brotto et al., 2010; Dawson et al., 2016; Higginbottom, 2024; Hille et al. (2024); Scherrer, 2010; Van Houdenhove et al., 2015). For asexual individuals, non-monogamous relationships may hold certain benefits, such as freeing them from perceived pressure to have sex and make compromises on physical intimacy, while still obtaining the emotional intimacy of a relationship (see also Copulsky, 2016; Hille et al., 2024). Of note, wanting a non-monogamous relationship is not mutually exclusive with wanting a monogamous relationship: Both relationship types can be wanted or not wanted at the same time, or preferred to varying degrees.

Dawson et al. (2016) noted that the most common form of intimacy that was mentioned during interviews with asexual individuals were friendships. Asexual individuals identifying as not being romantically interested (i.e., aromantic) tend to describe their ideal relationship as more friendship-like companionships (Scherrer, 2008). Accordingly, asexual individuals may prefer relationships that are committed but in a friendship-like companionship rather than in a traditional romantic way. Many long-term partners have children together, generally requiring heterosexual intercourse. Thus, it may not be surprising that asexual individuals are less likely to have children than allosexual individuals (Aicken et al., 2013; Greaves et al., 2017). In a study employing a sample of U.S. college students, Hall and Knox (2022) found lower interest in parenthood among asexual compared to heterosexual individuals. However, 57% of the asexual sample still indicated wanting to have children. Given the young average age (20 years) and limited demographic (college students) of the sample, more research with older and more diverse samples is needed to understand asexual individuals’ (dis-)interest in becoming a parent.

#### Ideal Long-Term Partner Preferences

There is also little known about the partner preferences of asexual individuals. Research on partner preferences in general showed that across cultures and genders, people strongly prefer a partner who is kind and supportive (e.g., Fletcher et al., 1999; Locke et al., 2020). Li et al. (2002) concluded that kindness in a partner is considered a necessity rather than a luxury.

Some evidence suggests that heterosexual women value high self-esteem and being socially dominant (i.e., confident, extraverted, authoritative) in a partner more than do heterosexual men (Botwin et al., 1997; Sadalla et al., 1987), possibly because these traits represent the “stereotypical male.” Physical attractiveness has also been seen as an important attribute in prospective romantic partners (e.g., Eastwick et al., 2014). According to some evolutionary theories, these characteristics are indicative of potential reproductive success (see Buss & Schmitt, 1993; Gangestad & Simpson, 2000). As asexual women lack or experience only little sexual attraction, they may put less emphasis on traits related to male stereotypes or a partner's sexual experience and physical attractiveness.

Interestingly, Scheller et al. (2023) found that individuals with reduced sexual attraction placed less importance on having a physically attractive partner, while Edge and Vonk (2024) did not find sexual orientation differences related to physical attractiveness. Edge and Vonk (2024) also did not find evidence for any sexual orientation differences in preferences for a partner who is intelligent, kind and understanding, has good earning capacity, and is a college graduate. However, these findings may be due to the relatively small sample size (*n* = 388 with 149 asexual individuals), constraining the power to detect smaller differences.

Importantly, it is likely that a person’s relationship interests influence what they look for in a partner. For example, people uninterested in long-term relationships are unlikely to place importance on the attributes of a potential long-term partner. Similarly, the intention to become a parent could possibly alter the importance of some traits (e.g., attractiveness or financial security). Thus, a consideration of the effects of relationship interests on current partner preferences could give a better picture of the partner preferences of asexual and heterosexual women.

Lastly, individuals seem to look for (and often have) a partner that is similar to themselves (e.g., Dijkstra & Barelds, 2008). This so-called similarity-attraction hypothesis gives rise to the question of how asexual individuals characterize themselves and whether their non-sexual characteristics substantially differ from those with other sexualities.

#### Personality

Two quantitative studies have looked into Big Six personality differences between asexual people and people identifying as other sexual orientations (Bogaert et al., 2018; Greaves et al., 2021).^1^ In both studies, asexual individuals scored lower on extraversion and higher on openness to experience than heterosexual individuals. Further, using propensity-score matched samples, Greaves et al. (2021) found lower agreeableness in asexual individuals (compared to heterosexual individuals). In addition, extraversion has been positively linked with assertiveness (Bouchard et al., 1988). Research suggests that asexual individuals tend to be more socially withdrawn (Brotto et al., 2010) and show less approach motivation in relationships than allosexual individuals (Greaves et al., 2021). Asexual individuals may thus be expected to describe themselves as less assertive and less confident.

Asexuality was found to be negatively associated with positive body image (Swami et al., 2019), which contrasts with the finding that body appreciation tends to be higher among other sexual minorities such as lesbian and bisexual women than heterosexual women (Ramseyer Winter et al., 2015) and suggests that asexual individuals may feel less pressure to be attractive to others (Swami et al., 2019). However, given the finding of asexual individuals having a more negative body image (Swami et al., 2019), asexual individuals may likely display other negative body perceptions such as feeling physically unattractive.

### The Current Study

While there has been an upsurge in research on asexuality, especially on the diverse experiences within the asexual spectrum (Hille et al., 2023), research comparing asexual and heterosexual preferences in relationships and partners is still scarce (Edge & Vonk, 2024; Scheller et al., 2023). However, understanding relationship and partner preferences when sexual attraction is reduced or absent is important, as it allows us to examine the full plethora of human (non-)sexuality. While previous studies have shed light on variation within the asexual spectrum on whether asexual individuals desire (romantic) relationships and if so, how they picture their ideal relationship, the goal of the present study was to investigate differences between self-identified asexual and heterosexual women in their preferred relationship options and ideal partner preferences for a long-term relationship. Additionally, we examined whether asexual and heterosexual women differ in their self-ratings on the same preference attributes.

To our knowledge, this is one of the first studies to directly test for differences between asexual and heterosexual women regarding their preferences in a long-term partner and for different types of relationships. We use data from a large (asexual *n* up to 390), multinational sample (including participants from 38 countries) recruited through a female health app to provide more reliable and generalizable results. To provide estimates that are more robust to confounding, we matched each asexual person with a heterosexual person using propensity score matching, thereby reducing confounding due to observed covariates. To account for unobserved confounding, sensitivity analyses were performed. Because our sample largely consisted of participants identifying as women, we focused our hypotheses and analyses exclusively on women.

Although previous research has found considerable heterogeneity among asexual individuals in how they picture their ideal relationship, one common denominator emerged: desiring a relationship that does not involve sexual intercourse (e.g., Dawson et al., 2016). Thus, we hypothesized that asexual women show lower interest in having sexual, non-romantic relationships (H1.1) and higher interest in non-sexual, romantic relationships than heterosexual women (H1.2). Research has found that non-monogamous relationships as well as alternative committed relationships (such as companion-like relationships) are possible relationship options for asexual individuals (e.g., Higginbottom, 2024). Hence, we hypothesized that asexual women show higher interest in non-monogamous relationships (H1.3) and higher interest in alternative committed relationships (H1.4) than heterosexual women. Considering the difficulties that arise with having a relationship for asexual individuals, including having to negotiate physical intimacy and potential aromantic tendencies (see Dawson et al., 2016), we hypothesized that asexual women show higher interest in being non-partnered than heterosexual women (H1.5).

Based on previous findings regarding partner preferences and drawing on the notion that asexual women, due to a lack of sexual attraction, may place less value on attributes that reflect male stereotypes as compared to heterosexual women, we expected that asexual women would show lower importance ratings than heterosexual women for a confident and assertive partner (H.2.1), a physically attractive partner (H2.2), and a sexually experienced partner (H2.3).

Individuals commonly want a partner that is similar to themselves (so-called similarity-attraction; Dijkstra & Barelds, 2008). In line with this notion, as well as our hypotheses on partner preferences, and prior research (Aicken et al., 2013; Bogaert, 2004; Greaves et al., 2021; Swami et al., 2019), we expected the following: Asexual women would rate themselves as lower in confidence and assertiveness (H3.1) as less attractive (H3.2), and as being less sexually experienced than heterosexual women (H3.3) (see supplemental material S1 for the exact wording of all preregistered hypotheses).

Whether asexual and heterosexual women differ in wanting a monogamous relationship and wanting to become a parent was evaluated in an exploratory manner.^2^ Furthermore, differences between asexual and heterosexual women in desiring a partner that is kind and supportive, educated and intelligent, and financially secure and successful was also explored. Similarly, differences in self-ratings on the same attributes were investigated. To control for potential influences of specific relationship interests on partner preferences, preference ratings were re-analyzed while matching participants on variables that indicate disinterest in long-term relationships, interest in purely sexual relationships, and parenting desire.

## Method

### Participants and Procedure

Data have been collected via the Ideal Partner Survey, in collaboration with the female health app Clue. Data from the Ideal Partner Survey was used by Botzet et al. (2023) to study the link between age and partner preferences, and by Kuschel et al. (2025) to examine the link between political orientation and partner preferences. The survey was advertised through emails sent to the users of Clue and myOne (a condom company) as well as through push messages within the Clue app. Advertisements were further broadcasted via social media channels such as Twitter, Facebook, and Instagram. The survey was set up via the software Typeform v2 (https://www.typeform.com/). Inclusion criteria were 18 or older and affirmative response that they had taken the survey seriously. Measures from the Ideal Partner Survey relevant for the current study are demographics, ideal long-term partner preferences, self-ratings, and interests for certain preferred relationship options (for more detailed information on the survey set up and procedure, see Botzet et al., 2023).

Because the data used in the current study were collected in collaboration with Clue, the large majority of participants identified as women. Although a condom company, MyOne, helped to recruit a sample of participants identifying as men, after data collection and imposing our exclusion criteria, only 1,535 (.03%) of the sample identified as men and 102 (.002%) identified as nonbinary/genderqueer. Of these, only *n* = 12 men and *n* = 68 nonbinary/genderqueer individuals identified as asexual. Given that these sample sizes are not comparable to our sample of women (*n* = 51,775, of which *n* = 447 identified as asexual), we decided to focus our analyses exclusively on women. However, to test the robustness of the findings, all analyses were run again including individuals identifying as men and nonbinary/genderqueer.^3^ It is important to emphasize that the few participants identifying as men or genderqueer/nonbinary may not be representative and may distort actual differences. Generalizations beyond women should be made very cautiously.

In addition, participants identifying as women who did not identify as asexual or heterosexual, and participants who indicated not having filled out the survey truthfully were excluded. The total sample size comprised 68,085 responses. After applying the exclusion criteria, a sample size of *n* = 51,775 remained. See supplemental material S2 for a flowchart of applied exclusion criteria.

After applying exclusion criteria, the unmatched sample consisted of a total of *n* = 51,775 participants (asexual: *n* = 447, heterosexual: *n* = 51,328). Asexual participants were on average 24.03 years old (*SD* = 6.56, range = 18–56) and heterosexual participants were on average 25.13 years old (*SD* = 7.30, range = 18–80). Participants came from 173 different countries.

#### Propensity Score Matching

To create comparable groups, each asexual individual was matched with a heterosexual individual using propensity score matching (Rosenbaum & Rubin, 1983). Propensity score matching finds “statistical twins” on certain covariates that may exert an influence on the outcome variables. Through this matching process confounding was minimized and thus confidence is higher that the effects solely stem from the differences in sexual orientation rather than from differences in certain covariates. This procedure allows for a more direct comparison between groups for which random allocation may not be feasible (here: sexual orientation). While observational data usually does not allow for causal inferences, propensity score matching approximates randomization, facilitating more causal interpretations based on specific assumptions. The covariates age, country, language, relationship status, and relationship length were used as matching variables.^4^ See Table 1 for the item wordings.

**Table 1:** All covariates relevant for the study

Variable	Scale/Item
Language^a^	Survey versions: English German Danish French Japanese Chinese Portuguese Spanish Russian Italian
Age	*How old are you?*
Country	*In what country do you live?*
Relationship status	*Select your relationships during the past 3 months* *Choose as many as you like* No romantic or sexual relationships during the past 3 months Short-term (casual) sexual relationship (e.g. hookups or one-night-stands) New (less than 1 month old) romantic and/or sexual relationship Ongoing (longer than 1 month) uncommitted/non-exclusive romantic and/or sexual relationship Long-term committed/exclusive sexual relationship with one or more partners Other
Relationship duration^b^	*How long have you been in this long-term relationship?* *Please answer in years*
Gender^c^	*Do you identify as…* Woman Genderqueer/Nonbinary Man None of the above Prefer not to say Other

^a^Language in which the survey was filled out. This variable was not assessed as an item but was coded based on the chosen survey version

^b^Only participants who indicated in an item before that they were in “long-term committed/exclusive sexual relationship with one or more partners” were asked about relationship duration. The relationship duration of all other participants was set to 0

^c^Gender was included as a covariate only for the robustness check

Certain parts of the survey were optional; therefore, sample sizes differed for the different analyses (partner preferences *n* = 47,136, self-ratings *n* = 45,444, and relationship options *n* = 38,058). To ensure well-matched samples, propensity score matching was performed separately for each of the three outcome sections. To achieve a good covariate balance, different matching algorithms were run and the one that resulted in the best covariate balance was selected. For comparability reasons, the algorithm that was run for all matchings was based on the algorithm selected and computed for the outcome section with the largest sample of asexual individuals (i.e., ideal partner preferences; *n* = 390).

Optimal matching, in which participants are matched minimizing the average absolute distance across all pairs, as well as nearest-neighbor-matching which selects the best matching partner for each asexual participant one at a time, were performed. The nearest-neighbor-matching algorithm has the option to include a caliper, a factor that limits how large the distance between propensity scores of matching partners is allowed to be. The caliper size was varied in size to see whether it brought improvement in covariate balance. Note that with nearest-neighbor-matching using a caliper, it is possible that asexual participants may be discarded.

The main criterion for assessing covariate balance was the standardized mean difference. Following the suggestion by Stuart (2010), the algorithm that produced the smallest standardized means across the largest number of covariates was selected. As a rule of thumb, standardized mean differences below the cut-off of 0.10 (Austin, 2011b) were considered small enough. The variance ratio of the two groups should approximate 1 after matching. Additionally, the area of common support, which represents the overlap of propensity score distributions of the unmatched groups, was inspected. A small overlap suggests that the effects are confined to only a specific subsample. In contrast, a high overlap suggests that the effects can be generalized to represent the whole of the sample (Thoemmes & Kim, 2011).

**Fig. 1:**
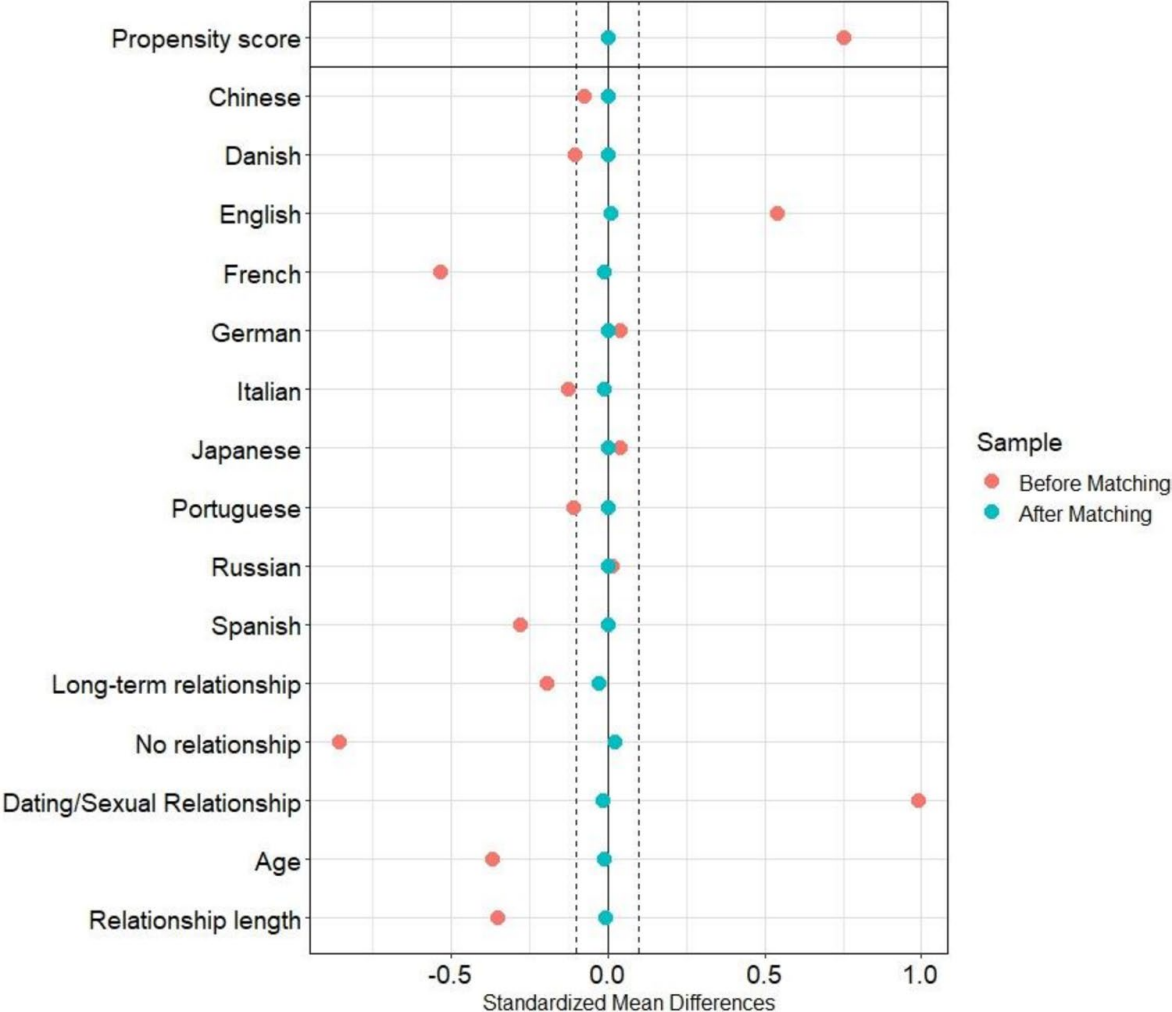
Standardized mean differences across all covariates before and after matching. The dotted vertical lines indicate the cut off of ( −)0.1 standardized mean difference (SMD). SMDs > 0 indicate that heterosexual women have higher mean scores. SMDs < 0 indicate that asexual women have higher mean scores. The country variables were omitted from the figure due to space restrictions and a clearer visualization. Nearest-neighbor-matching without caliper adjustment was run. Values are based on the partner preference sample (*N* = 780)

For the partner preference sample, the optimal matching algorithm resulted in 27 covariates not being balanced (i.e., standardized mean differences > 0.10). The nearest-neighbor-matching algorithm without any caliper adjustment resulted in all variables being balanced based on standardized mean differences (see Fig. 1). Thus, the nearest-neighbor-matching approach met the criteria set in the internal preregistration. Variance ratios were also close to 1 for age and relationship length, indicating that the ratios of variance in the asexual and heterosexual sample were similar after matching. The area of common support of the propensity scores was high, indicating that the two groups are likely to be representative for the whole sample (see also Figure S4 in supplemental material).

To see whether limiting the caliper width brought any improvement in balance properties (without losing asexual participants), nearest-neighbor matching was rerun with a caliper of 0.25 and 0.10.^5^ Both algorithms did bring about minor balance improvements in few covariates but lead to one and three asexual participants, respectively, not being matched. Nearest-neighbor-matching without caliper adjustment was consequently chosen as the algorithm for all analyses. For means and balance properties of the covariates in the two groups before and after matching see supplemental material S6.

After matching, the matched partner preference sample consisted of *n* = 780 participants, 390 asexual and 390 heterosexual women respectively. The matched self-rating sample consisted of 386 asexual and 386 heterosexual women (*n* = 772). And last, the matched relationship options sample consisted of 323 asexual and 323 heterosexual women (*n* = 646).^6^

Matched participants were on average 24.02 years old (*SD* = 6.50, range = 18–52). They were from 38 different countries. The largest groups came from the U.S. (28%, *n* = 181) and Germany (13%, *n* = 82; see Table S8 in the supplement for a complete list of countries, counts and percentages of participants per country). Of the matched pairs, 92% (*n* = 258) were from the same country. Half (*n* = 323) of the respondents selected the English version of the survey; other selected languages are given in Table S4 and include German (15%, *n* = 99), Spanish (12%, *n* = 74), French (7%, *n* = 43), Portuguese (5%, *n* = 35), Italian (4%, *n* = 28), Japanese (4%, *n* = 19), Danish (2%, *n* = 15), and Russian (2%, *n* = 10). Of the matched pairs, 96% (*n* = 376) had corresponding languages. Regarding relationship status, 25% (*n* = 164) were in a long-term relationship, 5% (*n* = 32) were dating/in a sexual relationship, and 70% (*n* = 450) had no relationship. Of those in a long-term relationship, the average duration was 1.18 years (*SD* = 3.35, range = 0–31). Table 2 provides a detailed summary of descriptives, displayed separately for the asexual, matched heterosexual, and unmatched heterosexual samples. For a detailed group comparison on all demographic variables, see Table S7 in the supplement.

**Table 2:** Means and standard deviations for asexual, matched Hheterosexual, and unmatched heterosexual samples on all variables

Variables	Asexual Individuals		Matched Heterosexual Individuals		Unmatched Heterosexual Individuals	
	*M*	*SD*	*M*	*SD*	*M*	*SD*
Covariates^a^						
Age	23.94	6.40	24.12	6.61	25.21	7.30
Gender						
Woman	100%	–	100%	–	100%	–
Relationship status						
No relationship	68.97%	–	69.74%	–	63.38%	–
Dating/Sexual relationship	25.64%	–	24.62%	–	22.82%	–
Long-term relationship	5.38%	–	5.64%	–	13.80%	–
Relationship length (in years)	1.16	3.32	1.18	3.38	2.33	4.35
Outcomes						
Preferred relationship options						
Sexual, non-romantic relationship(s)	0.66	1.33	1.73	1.83	1.52	1.79
Non-sexual, romantic relationship(s)	4.34	1.74	2.30	1.88	1.93	1.95
Monogamous relationship(s)	4.31	1.69	5.04	1.47	4.86	1.75
Non-monogamous relationship(s)	1.13	1.58	0.83	1.43	0.88	1.52
Alternative committed relationship(s)	4.50	1.66	3.42	2.11	3.08	2.18
Being non-partnered	3.33	1.89	2.25	1.66	1.81	1.73
Becoming a parent	2.34	2.19	3.99	2.08	3.86	2.13
Ideal partner preferences						
Confident-assertive	3.97	1.15	4.37	0.97	5.47	0.63
Attractive	3.15	1.54	3.85	1.15	3.88	1.19
Sexually experienced	1.37	1.85	2.62	1.93	2.98	1.93
Kind-supportive	5.44	0.71	5.54	0.55	5.47	0.63
Financially secure-successful	4.12	1.20	4.36	1.02	4.37	1.10
Educated-intelligent	4.91	1.03	4.89	1.03	4.92	0.98
Self-ratings						
Confident-assertive	3.24	1.31	3.77	1.15	3.95	1.13
Attractive	3.04	1.28	3.62	1.12	3.78	1.10
Sexually experienced	1.05	1.44	2.13	1.82	2.97	1.72
Kind-supportive	4.68	0.97	5.03	0.78	5.01	0.81
Financially secure-successful	3.39	1.16	3.90	1.06	3.94	1.06
Educated-intelligent	4.46	0.89	4.62	0.91	4.59	0.85

CI = confidence interval. The sample sizes for the asexual sample and the matched heterosexual sample were equal due to the matching process (i.e., for the relationship sample *n* = 323; for the partner preference sample *n* = 390; for the self-rating sample *n* = 386). The sample size for the unmatched heterosexual sample was *n* = 37,412 for the relationship sample, *n* = 46,356 for the partner preference sample, and *n* = 45,058 for the self-rating sample. Language and country variables are not presented in the table due to space restrictions

^a^Values for covariates are based on the partner preference sample. All other values are based on the respective samples for the specified outcome section

### Measures

#### Sexual Orientation

Participants indicated their current sexual orientation (single response) from a list (“Straight/Heterosexual”, “Lesbian/Gay/Homosexual”, “Bisexual/Pansexual”, “Queer”, “Asexual”, “Prefer not to say”, “Other”). Only those selecting straight/heterosexual or asexual were included in this study.^7^

#### Covariates

Item wordings are listed in Table 1. Participants were asked to indicate the gender they identify with (not biological sex), age, the country they live in, and the relationships they had in the past three months. Relationships were classified into three categories: (1) long-term relationship, (2) dating/sexual relationship, and (3) no relationship. If they reported being in a long-term committed/exclusive sexual relationship, they were asked to state the length of that relationship.^8^ Language was coded based on the chosen survey version. Ten parallel language versions of the survey were available: English, German, Danish, French, Japanese, Chinese, Portuguese, Spanish, Russian, and Italian. Translations were carried out by native speakers of each respective language. To ensure validity of the translations, a “four eyes” approach was undertaken, meaning one translator as well as one proofreader were responsible per language.

#### Outcomes

Outcomes are organized into three sections: seven preferred relationship option outcomes, five ideal long-term partner preference outcomes, and five self-rating outcomes. All measured variables and item wordings for preferred relationship options are summarized in Table 3. Items were assessed on a 7-point Likert scale ranging from 0 (*not at all interested*) to 6 (*very interested*). Variables and item wordings for ideal long-term partner preferences as well as self-ratings are displayed in Table 4. Again, items were rated on a 7-point Likert scale ranging from 0 (*not at all important*/*not at all*, respectively, for preference and self-ratings) to 6 (*very important*/*very much*). For the partner preference and the self-rating items the mean of two items was used to calculate each of the following dimensions: kind-supportive including the variables kind and supportive, attractiveness including attractive face and attractive body, financially secure-successful including financially secure and successful/ambitious, confident-assertive including confident and assertive, and educated-intelligent including educated and intelligent.

**Table 3:** Variables used for preferred relationship options

Variable	Scale/Item
	*How interested are you in each of the following relationship options at some time in the future?*^a^
Sexual, non-romantic relationship	Having sexual, non-romantic relationship(s) (e.g., hookups, one-night-stands)
Non-sexual, romantic relationship	Having romantic non-sexual relationship(s)
Monogamous relationship	Having monogamous relationship(s)
Non-monogamous relationship	Having non-monogamous relationship(s)
Alternative committed relationship	Having alternative committed relationship(s) (friendly, non-romantic, non-sexual companionships)
Being non partnered	Being non-partnered (e.g., solo, single, not committed)
Becoming a parent	Becoming a parent (e.g., having and/or adopting children, being a step-parent)

^a^All preferred relationship option items were measured on a scale ranging from 0 = *not at all interested* to 6 = *very interested*

**Table 4:** Variables used for partner preference and self-ratings

Dimension/Variable	Scale/Item	
	Partner preferences	Self-ratings
	*For each trait below, first tell us how important it is to you when choosing an ideal long-term partner.*^a^	*How would you DESCRIBE YOURSELF on the traits below?*^b^
Kind-supportive		
Kind	Kind—How important is it to you?	Kind
Supportive	Supportive—How important is it to you?	Supportive
Attractiveness		
Attractive body	Attractive body—How important is it to you?	Attractive body
Attractive face	Attractive face—How important is it to you?	Attractive face
Financially secure-successful		
Financially secure	Financially secure—How important is it to you?	Financially secure
Successful-ambitious	Successful/ambitious—How important is it to you?	Successful/ambitious
Confident-assertive		
Confident	Confident—How important is it to you?	Confident
Assertive	Assertive—How important is it to you?	Assertive
Educated-intelligent		
Intelligent	Educated—How important is it to you?	Educated
Educated	Intelligent—How important is it to you?	Intelligent
Sexually experienced	Sexually experienced—How important is it to you?	Sexually experienced

Except for the variable “sexually experienced,” dimensions are based on two variables each: e.g., kind-supportive is the mean of the variables kind and supportive

^a^All partner preference items were measured on a scale ranging from 0 = *not at all important* to 6 = *very important*

^b^All self-rating items were measured on a scale ranging from 0 = *not at all* to 6 = *very much*

### Statistical Analysis

There exists an ongoing debate about whether statistical analyses following propensity score matching should account for the paired nature of the data. Advocates for handling the data as independent refer to the fact that propensity score matching does not guarantee pairs to have equal values on all covariates, merely that within groups of individuals with similar propensity scores, the covariates will be distributed similarly (see Stuart, 2008). However, Austin (2011a) showed that methods for paired samples outperformed methods for independent samples using Monte Carlo simulations. Thus, following Austin’s (2011a) suggestion, the hypotheses were tested using dependent *t*-tests.

All 11 hypotheses are directional. Therefore, one-sided *t*-tests were carried out. Analyses were based on the respective propensity score-matched sample that was created for each outcome section. For each hypothesis, a dependent one-sided *t*-test was computed with sexual orientation (asexual or heterosexual) as the predictor variable and the outcome as specified in each hypothesis. All analyses were run using R Statistical Software v4.1.3 (R Core Team, 2022). We used the packages MatchIt v4.3.4 (Ho et al., 2011) and optmatch v0.10.0 (Hansen & Klopfer, 2006) for propensity score matching; rbounds v2.2 (Keele, 2022) and EValue v4.1.3 (Mathur et al., 2018) for sensitivity analyses; and forestplot v2.0.1 (Gordon & Lumley, 2021) and cobalt v4.3 (Greifer, 2022) to create figures.

#### Inference Criteria

The *p*-value was the main inference criterion. The significance threshold was set at .05. Since individual hypotheses were tested, correction for multiple testing did not have to be applied (see Rubin, 2021). Although not specified in the internal preregistration, in addition to statistical significance, effect sizes and their 95% confidence intervals were interpreted looking at the smallest effect size of interest (SESOI) by performing a test of practical equivalence (Lakens et al., 2018). Setting boundaries for a SESOI allows one to decide on the practical significance of an observed effect. There are three possible scenarios for which the SESOI gives decision guidance:

1. If the effect size and its 95% confidence interval fall completely outside the boundaries of the SESOI, the effect is accepted as substantial.
2. If the effect size and its 95% confidence interval neither are completely within nor completely outside the boundaries of the SESOI, evidence is inconclusive. It cannot be decided on whether the effect size is substantial or not and larger sample sizes are needed because the estimates are insufficiently precise.
3. If the effect size and its 95% confidence interval fall completely within the boundaries of the SESOI, the effect size is interpreted as not substantial (sometimes referred to as negligible).

Since not many quantitative studies have examined differences in preferred relationship options and partner preferences of asexual and heterosexual individuals and that even more specifically in a propensity score matched sample, we chose the study by Greaves et al. (2021) as a reference for the SESOI. To the best of our knowledge, this is the only study that employed propensity score matching to compare asexual and heterosexual individuals. However, Greaves et al. primarily looked at personality and thus generalizing these effect sizes to other domains such as partner preferences or relationship options can only be made with caution. The SESOI was based on the smallest significant effect size that was found by Greaves et al., which is *d* = 0.26 (for the personality dimensions honesty-humility and conscientiousness). Hence, the SESOI boundaries were set at *d* =  − 0.26 and *d* = 0.26. Note that those SESOI boundaries are more conservative than those commonly used (Lakens et al., 2018).

#### Robustness Checks

Robustness checks were performed running all preprocessing steps and statistical models including men and genderqueer/non-binary participants to account for potential differences in gender. For comparability, participants were matched using the same propensity score matching algorithm that was selected and used in the main analyses (i.e., nearest-neighbor-matching without caliper adjustment). For details on the matching procedure, see supplement S16–S18.

After matching, the partner preference sample consisted of *n* = 920 participants (*n* = 460 asexual individuals and *n* = 460 heterosexual individuals). Of these, *n* = 18 (2%) were men and *n* = 93 (10%) were genderqueer/nonbinary, and *n* = 809 (88%) were women. Seven covariates did not meet the preset criteria for balance.^9^ However, the standardized mean differences of all unbalanced covariates were < 0.25, which is still a good balance considering that our preset criterion, following Austin’s (2011b) suggestion of standardized mean differences < 0.10, is fairly conservative. Stuart (2010), for example, suggests that covariates with standardized mean differences < 0.25 can be considered balanced. For details on the other two analysis samples, see Table S16 in the supplement.

#### Sensitivity Analyses

To account for potential unobserved confounders not captured by the measured covariates (i.e., hidden bias), sensitivity analyses were performed to estimate how robust the results are to hidden bias. Although a sensitivity analysis cannot control for unobserved confounding, it quantifies how large the hidden bias would need to be to change the conclusions substantially. We performed Rosenbaum’s (2005) sensitivity analysis which finds the threshold at which the influence of the unobserved confounders would render the conclusions given the measured covariates inconclusive. Rosenbaum’s sensitivity analysis was the main criterion to evaluate how sensitive the study is to hidden bias.^10^ Sensitivity analyses were carried out only for statistically significant differences.

#### Exploratory Analyses

Exploratory analyses for differences on the dimensions kind-supportive, financially secure-successful, and educated-intelligent as well as for differences in interest in monogamous relationships and becoming a parent were carried out in the same manner as outlined above (i.e., using dependent *t*-tests). However, no predictions about the direction of effects were made beforehand. Thus, the tests were two-sided.

Arguably, there might be individuals who are not interested in a long-term relationship at all. The part of the survey that asked participants to indicate their preferences for a potential long-term partner was included in the mandatory part of the survey and thus could not be skipped by participants who do not care for long-term relationships. In reaction to this, it is possible that this general disinterest in long-term relationships is reflected by giving lower importance ratings to all partner preference items in general. Other relationship interests (such as a general disinterest in sexual relationships or a disinterest in parenting) could also potentially influence partner preference ratings.

To control for the influence of relationship interests on partner preferences, three additional exploratory analyses were carried out. First controlling for disinterest in long-term relationships and then additionally controlling for either interest in purely sexual relationships or parenting desire while holding disinterest in long-term relationships constant.

To solely control for disinterest in long-term relationships, participants were re-matched based on the variables interest in monogamous relationships, interest in non-sexual, romantic relationships, and interest in being non-partnered in addition to the covariates that were used for matching in the main analyses.

To additionally control for interest in purely sexual relationships, the variable interest in sexual, non-romantic relationships (e.g., hookups) was included in the matching process besides the variables used while controlling for disinterest in long-term relationships. Similarly, to additionally control for parenting desire, the variable interest in becoming a parent was included in the matching process besides the matching variables used for controlling for disinterest in long-term relationships. After matching, dependent *t*-tests were calculated in the same manner as explained above.

### Availability of Data and Code

All code (for data wrangling and statistical analyses) is accessible via the corresponding project on the Open Science Framework (https://osf.io/m4c8f/) and on https://github.com/paulabange/idealpartnersurvey_asexuality_psm. Due to the sensitive nature of the data, it is not possible to make it publicly available. However, based on Quintana’s (2020) primer for synthetic datasets, we created a synthetic dataset that mimics many of the features of the real data such as means and bivariate associations. This synthetic dataset was uploaded to the Open Science Framework and thus can be used by others to run the code pertaining to this project with a realistic fake dataset.

## Results

The relative ranking of the relationship options varied markedly between asexual and heterosexual women. For example, while for asexual women, alternative committed relationships ranked highest and sexual, non-romantic relationships ranked lowest, for heterosexual women, monogamous relationships ranked highest and non-monogamous relationships ranked lowest. The relative ranking of partner preference attributes was similar across asexual and heterosexual women. Consistent with previous research, kindness and supportiveness in a partner were valued most by both asexual and heterosexual women, followed by education and intelligence. Financial security and successfulness, confidence and assertiveness, as well as attractiveness ranked in the midfield. A sexually experienced partner ranked lowest in importance. Rankings for the self-ratings were the same across both sexual orientation groups and very similar to the ranking of the partner preferences. Descriptives for all variables, including covariates, preferred relationship options, ideal partner preferences, and self-ratings, are shown in Table 2.

Associations between interest in different relationship options showed a nuanced pattern. Reflecting a more conventional view of relationships, interest in monogamous relationship(s) correlated positively with interest in becoming a parent (*r* = .28), while both—interest in monogamous relationship(s) and interest in becoming a parent—correlated negatively with interests in non-monogamous relationship(s), alternative committed relationship(s), and being non-partnered" (*r*s ranging from − .08 to − .36). On the flipside and mirroring openness to more alternative ways of living, interests in having non-sexual, romantic relationship(s), non-monogamous relationship(s), and alternative committed relationship(s) as well as interest in being non-partnered all correlated positively with each other (*r*s ranging from .12 to .30). Finally, being interested in sexual, non-romantic relationship(s) correlated positively with interest in having non-monogamous relationship(s) (*r* = .28). Almost all partner preference outcomes were positively correlated (*r*s ranging from .10 to .39), except for the correlations between the preference for kindness-supportiveness and the preference for attractiveness (*r* = .05) as well as the preference for kindness-supportiveness and the preference for sexual experience (*r* = .004). Preferences for confidence-assertiveness and financial security-successfulness showed the strongest correlation (*r* = .39). All self-rating outcomes were positively correlated, with the weakest correlation for the self-rating of confidence-assertiveness and the self-rating of kindness-supportiveness (*r* = .20) and the strongest correlation for the self-ratings of financial security-successfulness and education-intelligence (*r* = .54). Zero-order correlations between all outcome variables are shown in Table 5.^11^

**Table 5:** Zero-order correlations between all outcome variables

Variable	Preferred relationship options							Ideal long-term partner preferences						Self-ratings				
	1	2	3	4	5	6	7	8	9	10	11	12	13	14	15	16	17	18
Preferred relationship options																		
1. Sexual, non-romantic relationship(s)																		
2. Non-sexual, romantic relationship(s)	-.14**																	
3. Monogamous relationship(s)	.02	-.06																
4. Non-monogamous relationship(s)	.28**	.16**	-.19**															
5. Alternative committed relationship(s)	.10*	.30**	-.08*	.20**														
6. Being non-partnered	.03	.25**	-.29**	.12**	.25**													
7. Becoming a parent	.08	-.15**	.28**	-.08*	-.12**	-.36**												
Partner preferences																		
8. Confident-assertive	.13**	-.13**	.08*	-.02	-.06	-.08	.08											
9. Attractive	.14**	-.15**	.12**	-.07	-.12**	-.10*	.10*	.27**										
10. Sexually experienced	.36**	-.30**	.08	.05	-.16**	-.09*	.06	.30**	.31**									
11. Kind-supportive	-.08	.01	.16**	-.02	.08	-.12**	.19**	.11**	.05	.00								
12. Financially secure-successful	.09*	-.10*	.09*	-.05	.00	-.07	.06	.39**	.28**	.23**	.10**							
13. Educated-intelligent	.08	-.01	.11*	-.02	.08	.04	-.03	.32**	.27**	.16**	.17**	.37**						
Self-ratings																		
14. Confident-assertive	.10*	-.13**	.06	-.03	-.06	-.07	.14**	.53**	.25**	.22**	.10**	.25**	.25**					
15. Attractive	.09*	-.13**	.18**	.01	-.07	-.10*	.16**	.28**	.42**	.22**	.10**	.16**	.20**	.49**				
16. Sexually experienced	.25**	-.30**	.07	.09*	-.14**	-.15**	.06	.24**	.17**	.55**	.08*	.10**	.14**	.29**	.32**			
17. Kind-supportive	.00	-.05	.17**	-.02	-.01	-.22**	.26**	.13**	.04	.11**	.42**	.08*	.10*	.20**	.26**	.22**		
18. Financially secure-successful	.08	-.14**	.16**	-.02	-.09*	-.15**	.21**	.28**	.19**	.13**	.10*	.35**	.26**	.46**	.43**	.24**	.26**	
19. Educated-intelligent	.09*	-.06	.16**	-.03	.06	-.06	.05	.25**	.13**	.12**	.13**	.12**	.46**	.41**	.39**	.21**	.27**	.54**

*n* = 944. Note that sample sizes may differ for each reported correlation due to missings

*p < .05

**p < .01

**Fig. 2:**
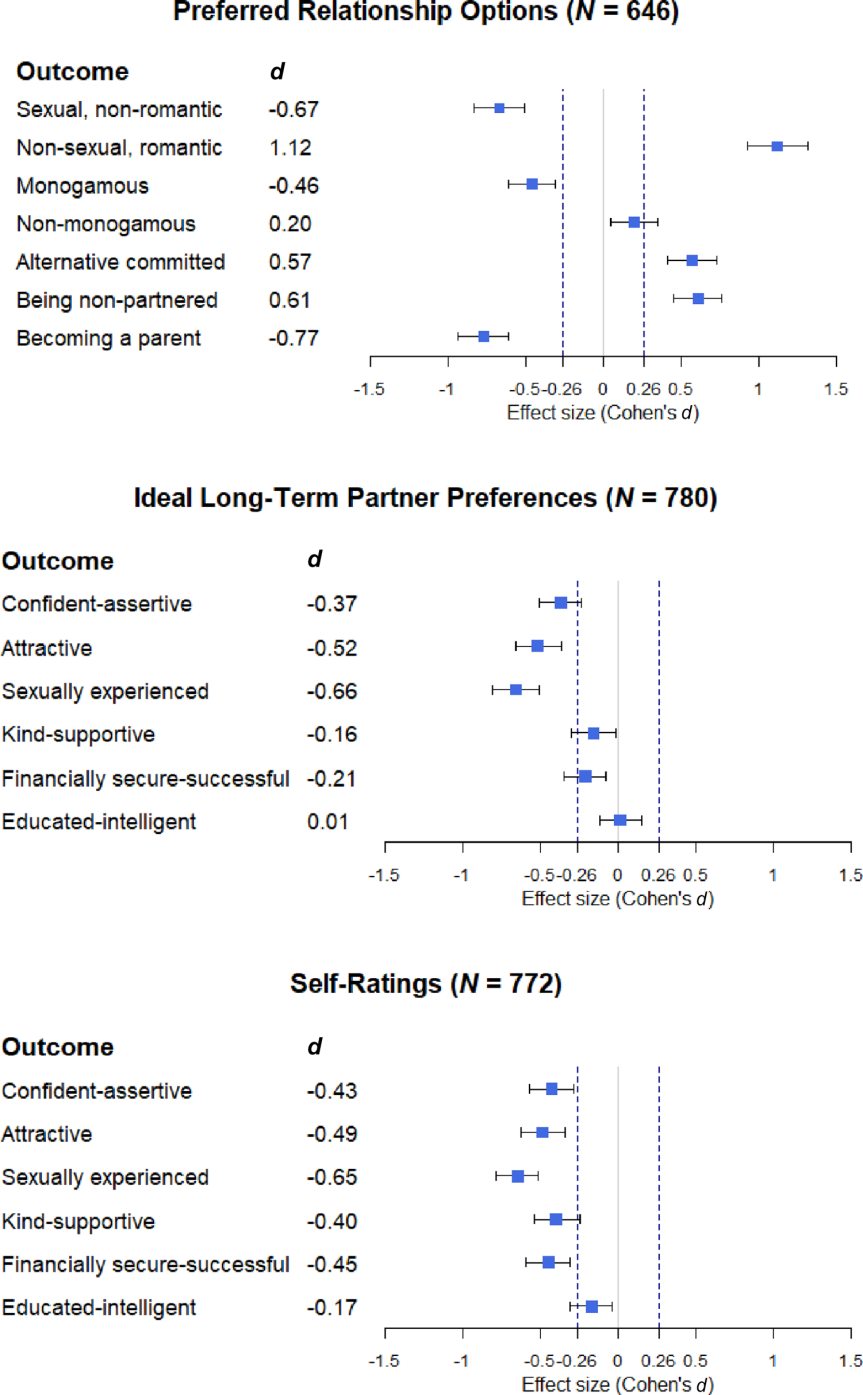
Effect sizes and 95% Confidence Intervals for all outcomes. Effect sizes (Cohen’s *d*s) with their 95% confidence interval are displayed. Vertical dotted lines indicate the SESOI boundaries (*d* = (−)0.26)

Gauging whether an effect size may be of practical relevance requires more than merely interpreting statistical significance (Lakens et al., 2018). Thus, the following sections will interpret the findings both in terms of statistical significance as well as their effect sizes and the predefined SESOI boundaries. Results for all *t*-tests are summarized in Table 6. Figure 2 displays the effect sizes including their 95% confidence intervals in relation to the SESOI boundaries for all outcome variables. In sum, considering only statistical significance with an alpha-level of .05, all hypotheses are supported. However, while most effect size estimates are substantial (H1.1; H1.2; H1.4; H1.5; H2.2; H2.3), for two hypotheses the evidence remains inconclusive (H1.3; H2.1). In addition, based on exploratory analyses, we found further evidence for substantial effects. We provide details on the results below.

**Table 6:** Results of dependent *t*-tests for all outcome variables

Outcome	Asexual Individuals		Heterosexual Individuals		*t*	*df*	*p*	*d*	95% CI
	*M*	*SD*	*M*	*SD*					
Preferred relationship options (*n* = 646)						322			
Sexual, non-romantic relationship(s)	0.66	1.33	1.73	1.83	− 8.96		< .001	** − 0.67**	[− 0.83, − 0.51]
Non-sexual, romantic relationship(s)	4.34	1.74	2.30	1.88	14.44		< .001	**1.12**	[0.93, 1.32]
Monogamous relationship(s)	4.31	1.69	5.04	1.47	− 6.11		< .001	** − 0.46**	[− 0.61, − 0.31]
Non-monogamous relationship(s)	1.13	1.58	0.83	1.43	2.58		.005	0.20	[0.05, 0.35]
Alternative committed relationship(s)	4.50	1.66	3.42	2.11	7.58		< .001	**0.57**	[0.41, 0.73]
Being non-partnered	3.33	1.89	2.25	1.66	8.27		< .001	**0.61**	[0.45, 0.76]
Becoming a parent	2.34	2.19	3.99	2.08	− 10.29		< .001	** − 0.77**	[− 0.94, − 0.61]
Ideal partner preferences (*n* = 780)						389			
Confident-assertive	3.97	1.15	4.37	0.97	− 5.75		< .001	− 0.37	[− 0.51, − 0.24]
Attractive	3.15	1.54	3.85	1.15	− 7.32		< .001	** − 0.52**	[− 0.66, − 0.37]
Sexually experienced	1.37	1.85	2.62	1.93	− 9.73		< .001	** − 0.66**	[− 0.81, − 0.51]
Kind-supportive	5.44	0.72	5.54	0.55	− 2.28		.02	− 0.16	[− 0.30, − 0.02]
Financially secure- successful	4.12	1.20	4.36	1.02	− 3.06		.00	− 0.21	[− 0.35, − 0.08]
Educated-intelligent	4.91	1.03	4.89	1.03	0.21		.83	0.01	[− 0.12, 0.15]
Self-ratings (*n* = 772)						385			
Confident-assertive	3.24	1.31	3.77	1.15	− 6.30		< .001	** − 0.43**	[− 0.57, − 0.29]
Attractive	3.04	1.28	3.62	1.12	− 7.00		< .001	** − 0.49**	[− 0.63, − 0.34]
Sexually experienced	1.05	1.44	2.13	1.82	− 10.72		< .001	** − 0.65**	[− 0.79, − 0.52]
Kind-supportive	4.68	0.97	5.03	0.78	− 5.70		< .001	− 0.40	[− 0.54, − 0.25]
Financially secure-successful	3.39	1.16	3.90	1.06	− 6.45		< .001	** − 0.45**	[− 0.60, − 0.31]
Educated-intelligent	4.46	0.89	4.62	0.91	− 2.54		.01	− 0.17	[− 0.31, − 0.04]

Sample sizes are in brackets. Substantial effect estimates are in bold. Scale ranged from 0–6

### Preferred Relationship Options

As expected, asexual women had significantly less interest in having sexual, non-romantic relationships than heterosexual women (a substantial effect of *d* =  − 0.67, 95% CI [− 0.83, − 0.51]; H1.1). Also, in line with the hypotheses, asexual women had significantly more interest in non-sexual, romantic relationships (a substantial effect of *d* = 1.12 [0.93, 1.32]; H1.2), non-monogamous relationships (an inconclusive effect of *d* = 0.20 [0.05, 0.35]; H1.3), alternative committed relationships (a substantial effect of *d* = 0.57 [0.41, 0.73]; H1.4), and being non-partnered (a substantial effect of *d* = 0.61 [0.45, 0.76]; H1.5).

Exploratory analyses showed that compared to heterosexual women, asexual women also had significantly less interest in monogamous relationships (a substantial effect of *d* =  − 0.46 [− 0.61, − 0.31]) and in becoming a parent (a substantial effect of *d* =  − 0.77 [− 0.94, − 0.61]).

### Ideal Long-Term Partner Preferences

Regarding the preferences for an ideal long-term partner, supporting the hypotheses, a confident-assertive (an inconclusive effect of *d* =  − 0.37, 95% CI [− 0.51, − 0.24]; H2.1), attractive (a substantial effect of *d* =  − 0.52 [− 0.66, − 0.37]; H2.2), and sexually experienced (a substantial effect of *d* =  − 0.66 [− 0.81, − 0.51]; H2.3) partner was less important to asexual women than to heterosexual women.^12^

In addition, exploratory analyses revealed that for asexual women, a partner that is kind-supportive (an inconclusive effect of *d* =  − 0.16 [− 0.30, − 0.02]) and financially secure-successful (an inconclusive effect of *d* =  − 0.21 [− 0.35, − 0.08]) was also less important than for heterosexual women. Only preference for an educated-intelligent partner showed no significant difference between asexual and heterosexual women (a non-substantial effect of *d* = 0.01 [− 0.12, 0.15]).

### Self-Ratings

Rating themselves on the same attributes, asexual women showed lower ratings on all six attributes than heterosexual women. As predicted, asexual women rated themselves lower in being confident-assertive (a substantial effect of *d* =  − 0.43, 95% CI [− 0.57, − 0.29]; H3.1), in being attractive (a substantial effect of *d* =  − 0.49 [− 0.63, − 0.34]; H3.2), and in being sexually experienced (a substantial effect of *d* =  − 0.65 [− 0.79, − 0.52]; H3.3).

Investigating differences on the other attributes in an exploratory manner showed that asexual women also rated themselves lower in being kind-supportive (an inconclusive effect of *d* =  − 0.40 [− 0.54, − 0.25]), in being financially secure-successful (a substantial effect of *d* =  − 0.45 [− 0.60, − 0.31]), and in being educated-intelligent (an inconclusive effect of *d* =  − 0.17 [− 0.31, − 0.04]).

### Item-Level Analyses

Each dimension (except for sexually experienced) was made up of two items. To check whether results were still consistent when looking at each item separately, differences between heterosexual and asexual women on each item were assessed. Performing item-level analyses for each dimension revealed no significant difference in estimates between items of the same dimension. As anticipated, no opposing effect directions were observed within each dimension supporting the results of the main analysis. See Table S13 in the supplement for a summary of results.

### Lifetime Sexual Partners and Sexual Experience

As would be expected, asexual and heterosexual women differed significantly in their number of lifetime sexual partners (*p* < .001, *t* =  − 5.10, *d* =  − 0.37, 95% CI [− 0.51, − 0.23]). While asexual women had an average of 1.82 sexual partners (*SD* = 4.85, median = 0, range = 0–53), heterosexual women had an average of 4.36 sexual partners (*SD* = 8.43, median = 1, range = 0–65). The total number of lifetime sexual partners was significantly and positively correlated with self-rated sexual experience (*r* = .52 [.47, .57]). In comparison, the unmatched heterosexual participants had on average 6.23 lifetime sexual partners (*SD* = 10.80, median = 3, range = 0–200), indicating that through matching heterosexual participants that are more sexually active were excluded.^13^

### Controlling for Relationship Interests

It might be argued that certain relationship interests, such as disinterest in long-term relationships, could influence partner preference ratings and possibly explain the overall lower preference ratings by asexual women. Controlling for disinterest in long-term relationships, the effects did not disappear, but rather the pattern became more pronounced. Notably, the effect for financially secure-successful more than doubled in size and became substantial (*d* =  − 0.50, 95% CI [− 0.66, − 0.33], original analysis: inconclusive effect of *d* =  − 0.21 [− 0.35, − 0.08]). The effect for confident-assertive also became substantial (*d* = 0.45 [− 0.61, − 0.30], original analysis: inconclusive effect of *d* =  − 0.37, [− 0.51, − 0.24]). Extending the matching model with controlling for interest in purely sexual relationships revealed that the effect for the preference for a sexually experienced partner decreased notably and thus could not be deemed substantial any longer (*d* =  − 0.37 [− 0.52, − 0.22], original analysis: substantial effect of *d* =  − 0.66, [− 0.81, − 0.51]). Extending the matching model instead with controlling for parenting interest shows a potential increase in effect size for financially secure-successful (inconclusive effect of *d* =  − 0.33 [− 0.49, − 0.17]) as compared to the original analysis (inconclusive effect of *d* =  − 0.21 [− 0.35, − 0.08]). For a summary of these additional analyses, see the supplement S14–S15.

### Robustness Check

To test the robustness of the results, all analyses were run again including men and genderqueer/nonbinary individuals besides women. Inspecting the confidence intervals of the effect estimates of the robustness analysis revealed that they always included the point estimate and/or confidence interval found in the main analysis. Thus, results did not change significantly when including other genders besides women. However, men and genderqueer/nonbinary individuals were underrepresented in the sample (88–89% women, 2% men, 9–10% genderqueer/nonbinary) and it was not possible to find a matching “twin” for every genderqueer/nonbinary asexual which led to several gender mismatches with heterosexual women. Thus, generalizations are limited and should only be made very cautiously. For a summary of the results of the robustness check, see the supplement S19–S20.

### Sensitivity Analyses

To test how sensitive the results are to potential unmeasured confounding, sensitivity analyses were performed. The following section summarizes the sensitivity results only for substantial effect estimates (based on the SESOI). For a summary of the results of the sensitivity analyses for all significant estimates, see Table 7.

**Table 7:** Rosenbaum bounds for all statistically significant results

Outcome	Γ^a^
Preferred relationship options (*n* = 646)	
Sexual, non-romantic relationship(s)	3.1
Non-sexual, romantic relationship(s)	5.3
Monogamous relationship(s)	2.1
Non-monogamous relationship(s)	1.2
Alternative committed relationship(s)	2.3
Being non-partnered	2.5
Becoming a parent	3.2
Ideal partner preferences (*n* = 780)	
Confident-assertive	1.7
Attractive	2.0
Sexually experienced	2.8
Kind-supportive	1.1
Financially secure/successful	1.2
Self-ratings (*n* = 772)	
Confident-assertive	1.8
Attractive	2.0
Sexually experienced	3.6
Kind-supportive	1.7
Financially secure-successful	1.9
Educated-intelligent	1.2

Sample sizes are given in brackets

^a^Γ = critical odds of differential sexual orientation due to unobserved confounding

Rosenbaum’s sensitivity analysis revealed that the magnitude of hidden bias that would render the substantial conclusions non-significant varied markedly across the different relationship option outcomes (Γ = 2.1–5.3), the ideal partner preference outcomes (Γ = 2.0–2.8), and the self-rating outcomes (Γ = 1.8–3.6). Taking the self-rating of confident-assertive, the outcome with the highest sensitivity to hidden bias (Γ = 1.8), as an example, this means, that if the odds of one person being asexual are only 1.8 times higher than the odds of another person with a similar propensity score due to unobserved confounding, the *p*-value for the comparison of self-rated confidence-assertiveness is no longer below the significance threshold of .05. In other words, even comparably small unobserved confounding effects could explain the substantial difference in self-rated confidence-assertiveness between asexual and heterosexual individuals.

For interest in non-sexual, romantic relationships, the outcome with the lowest sensitivity to hidden bias (Γ = 5.3), this means that even if the odds of one person being asexual are 5.2 times higher than the odds of another person with a similar propensity score due to unobserved confounding, this would still not render the *p*-value for the comparison of interest in non-sexual, romantic relationships non-significant. In other words, only comparably large unobserved confounding effects could explain the substantial difference in interest in non-sexual, romantic relationships between asexual and heterosexual individuals.

Taken together, the sensitivity to hidden bias appears to vary profoundly between the different outcomes, making it more likely that some of the observed results could be explained by unobserved confounding (mainly self-ratings: e.g., self-rating of confidence-assertiveness; self-rating of financially security-successfulness; all Γs < 2.0) than others (mainly relationship interests: e.g., interest in sexual, non-romantic relationship(s); interest in non-sexual, romantic relationship(s); interest in becoming a parent; and self-ratings of sexual experience; all Γs > 3.0).

## Discussion

This study used data from the Ideal Partner Survey to compare propensity score matched samples of self-identified asexual and heterosexual women on their preferred relationship options, ideal long-term partner preferences, and personality. As hypothesized, asexual women showed higher interest in having non-sexual, romantic relationships, non-monogamous relationships, alternative committed relationships, and being non-partnered and lower interest in having sexual, non-romantic relationships than heterosexual women (H1.1–1.5). They also showed lower interest in having monogamous relationships and in becoming a parent.

Generally, as compared to heterosexual women, asexual women gave lower importance ratings for the preference dimensions as well as for self-ratings of the same personal attributes. Consistent with hypotheses, asexual women attached less importance to an ideal long-term partner being confident and assertive, physically attractive, and sexually experienced (H2.1–2.3). Asexual women also attached less importance to a partner being kind and supportive as well as financially secure and successful, but no significant or substantial differences were observed for a partner being educated and intelligent. As expected, asexual women rated themselves lower in confidence and assertiveness, attractiveness, and sexual experience (H3.1–3.3). They also rated themselves lower in kindness and supportiveness, financial security and successfulness, and education and intelligence.

### Preferred Relationship Options

#### Interest in Sexual, Non-Romantic and Non-Sexual, Romantic Relationships

Findings for preferred relationship options clearly showed that many asexual women want (romantic) relationships (H1.2), which supports findings from qualitative studies (e.g., Dawson et al., 2016; Scherrer, 2008). Yet asexual women were found to be less interested in sexual, non-romantic relationships than heterosexual women (H1.1) and the difference is unlikely to be explained by unobserved confounding. This aligns with the definition of asexuality, namely, a lack of (or reduced) sexual attraction, as well as with previous studies showing that wanting a relationship without a sexual component seems to be at the core of asexuality (e.g., Brotto et al., 2010; Van Houdenhove et al., 2015). Consequently, asexual individuals are less inclined to pursue purely sexual relationships such as hookups and one-night stands. The largest observed difference emerged for interest in non-sexual, romantic relationships and this difference is also unlikely to be explained by unobserved confounding. While heterosexual women showed no interest in purely romantic relationships without a sexual component, asexual women were more interested in purely romantic relationships which again emphasizes the tendency for asexual individuals to favor romantic over sexual relationships (Van Houdenhove et al., 2015). This also supports the notion that romantic and sexual attraction are two distinct dimensions (see Diamond, 2003).

#### Interest in Non-Monogamous and Monogamous Relationships

As anticipated, asexual women were significantly more interested in non-monogamous relationships than heterosexual women (H1.3). However, this difference was small, and it remained inconclusive whether a substantial effect exists. Although non-monogamy can have benefits for asexual individuals, such as freeing them from engaging in sexual activities because the partner can be sexually active outside the partnership (Brotto et al., 2010; Copulsky, 2016; Hille et al., 2024; Van Houdenhove et al., 2015), there are also challenges to this type of relationship. Effective communication among partners is critical in managing relational processes (e.g., jealousy) in non-monogamous relationships (Gupta et al., 2024). Further, there may be unique challenges for asexual individuals, as engaging in a non-traditional relationship can amplify stigma and discrimination beyond and above that which may already be experienced by asexual individuals (MacInnis & Hodson, 2012). Overall, the low mean interest ratings for non-monogamy by both asexual and heterosexual women suggest that interest for this relationship option is rather low compared to other options.

Conversely, interest in monogamous relationships, which was investigated exploratorily, was relatively high for both sexualities. This indicates that monogamous relationships may be preferred over non-monogamous relationships not only by heterosexual individuals but also by asexual individuals. Even though interest was high for both groups, heterosexual women endorsed monogamous relationships substantially more than asexual women. One explanation for this discrepancy could be that asexual individuals who identify as aromantic are neither interested in non-monogamous relationships nor in monogamous relationships (Carvalho & Rodrigues, 2022), thereby downward biasing asexual women’s mean rating.

#### Interest in Alternative Committed Relationships

As hypothesized, interest in alternative committed relationships, in particular, friendly, non-romantic, non-sexual companionships, was substantially higher in asexual than heterosexual women (H1.4). This was in fact the highest rated relationship option among asexual women, which dovetails with the finding that friendships are an important form of intimacy for asexual individuals (Dawson et al., 2016). Thus, these types of relationships may substitute for romantic relationships. Arguably, there is heterogeneity in how asexual individuals define friendships and relationships. For example, Scherrer (2010) reported that while one participant described romantic relationships as non-sexual but “'deeper' than friendships” (p. 155), another noted that for them friendship and romantic relationship are two ends of a spectrum. Relationships that are committed and emotionally intimate yet non-sexual and non-romantic can take on varied forms and have also been referred to as queer platonic relationships (Higginbottom, 2024).

Such alternative relationship types appear to be a possibility for asexual women to have a committed, friendship-like relationship without feeling pressured to engage in sexual activities. For aromantic asexual individuals, these relationships may offer an opportunity to have a committed relationship with neither sexual nor romantic component (Carrigan, 2011; Higginbottom, 2024; Scherrer, 2008).

#### Interest in Being Non-Partnered

Asexual women were substantially more interested in being single (H1.5). One reason for asexual individuals to reject any type of relationship could be an aromantic orientation (Carvalho & Rodrigues, 2022). Alternatively, being single might allow asexual women to avoid difficulties that arise when seeking a partner and maintaining a relationship (Higginbottom, 2024). These may be particularly evident for asexual individuals when they are in a relationship with a sexual person having to negotiate and compromise on any form of intimacy (see Dawson et al., 2016).

#### Interest in Becoming a Parent

The exploratory finding that asexual individuals are substantially less interested in becoming parents is unlikely to be explained by unobserved confounding and dovetails with recent research (Hall & Knox, 2022). Indeed, asexual individuals are less likely to have children (Aicken et al., 2013; Greaves et al., 2017) which is also in accordance with other sexual minorities such as lesbians and gays being less likely to have children (Greaves et al., 2017). This does not imply that all asexual women are disinterested in having children. While they may be, on average, less interested in becoming a parent than heterosexual women, asexual women’s mean interest in parenting was not far away from the middle of the response scale, and the comparably large standard deviation implies that a number of asexual women would indeed be interested to become parents. For example, 15% of asexual women (vs. 36% of heterosexual women) indicated being very interested in becoming parents (the highest possible response anchor). This is in line with qualitative research showing that some asexual individuals do explicitly express the desire to become parents (Brotto et al., 2010).

Although desire to parent was generally lower among sexual minority individuals, Gates et al. (2007) reported that 41– 52% of childless gay and lesbian individuals indicated wanting to be a parent. In a study by Riskind and Patterson (2010), parenthood was even valued equally by gay/lesbian and heterosexual individuals. This suggests that many sexual minority individuals do want to become parents but that there are certain barriers to achieving parenthood. For example, asexual individuals are less likely to be in a relationship (e.g., Bogaert, 2004) and therefore lacking a partner to have (and raise) a child with. Gato et al. (2017) identified other barriers that sexual minorities may face such as financial costs for adoption, foster care, or reproductive technologies, but also openness about one’s sexual minority status, access to sexual minority community resources, and social support. Experiencing or anticipating these barriers may dampen the initial desire of sexual minority individuals for parenthood and consequently the intention to pursue it. This may even be more of a challenge for asexual individuals as asexuality along with the adversities that accompany it has not been as visible in the public as other sexual minorities.

### Ideal Long-Term Partner Preferences

All hypotheses were supported based on statistical significance. A partner who is confident and assertive was significantly less important to asexual women (H2.1). However, it remains inconclusive whether this effect should be considered substantial or not. Previous research found that heterosexual women prefer a partner who holds traits that signal social dominance (Botwin et al., 1997; Sadalla et al., 1987). These stereotypical male attributes may be evolutionarily indicative of a potential partner’s abilities to gain status and acquire resources to support a family (Buss & Schmitt, 1993; Gangestad & Simpson, 2000).

Differences in preferences for an attractive (H2.2) and a sexually experienced partner (H2.3) were significant and substantial. We hypothesized that because asexual women lack (or have reduced) sexual attraction, they would show lower preferences for physical attractiveness and sexual experience as compared to heterosexual women. Indeed, heterosexual women preferred an attractive partner more so than asexual women. This is in accordance with the results by Scheller et al. (2023) that physical attractiveness was less important for individuals with reduced sexual attraction. From an evolutionary viewpoint, physical attractiveness may be desirable in a partner because it has been suggested to be an indicator of health and viability (attributes that could also be passed on to subsequent children; Fletcher et al., 1999). In addition, asexual women showed a substantially lower preference for a sexually experienced partner than heterosexual women. This intuitively makes sense as asexual individuals lack (or have reduced) sexual attraction to any gender and have been shown to prefer to not engage in sexual activities (e.g., Dawson et al., 2016), rendering previous sexual experience of a partner less relevant.

We also found differences in exploratory analyses. In line with previous research on overall importance of partner preference attributes, kindness and supportiveness were valued most by both asexual and heterosexual women (e.g., Fletcher et al., 1999). In fact, the ranking of attributes by importance was almost identical for both sexualities suggesting that there is a large overlap in preferences. Although not substantial, a small significant effect was observed for preference for a kind and supportive partner. Asexual women preferred these attributes in a partner slightly less. This lack of a substantial difference is not surprising as kind-supportive was the highest rated dimension by both asexual and heterosexual women. Intelligence and education were highly valued by both asexual and heterosexual women; no significant or substantial difference was observed. These findings correspond with previous research reporting no sexual orientation differences in preferences for these attributes (Edge & Vonk, 2024). Kindness, supportiveness, intelligence, and education appear to be almost universally desired in a partner (see also Fletcher et al., 1999; Gerlach et al., 2019).

Asexual women prioritized a financially secure and successful partner less than heterosexual women. Financial security may be an important attribute in a partner that one wants to raise a child with. From an evolutionary perspective, resources and status may be indicative of someone’s capability of sustaining and supporting a family (Fletcher et al., 1999). Indeed, in a study covering more than a decade, individuals who had children over the study period, increased in preferences for status and resources in a partner (Driebe et al., 2024). Asexual women de-prioritizing financial security and successfulness then dovetails with the result that asexual women are less interested in becoming parents: When asexual individuals are less interested in becoming parents, the desire for a partner who can financially support them is likely to be lower as well. However, the effect for financial security-successfulness was inconclusive and thus cannot be interpreted as substantial or not. Future studies with larger sample sizes are needed.

Sexual reproduction, which usually requires heterosexual intercourse, is at the core of evolutionary accounts of partner preferences (e.g., Gangestad & Simpson, 2000). Our findings indicate that when sexual attraction is lacking (or reduced), attributes that are linked to sexual reproduction and raising children are less prioritized in a long-term partner. This suggests that in the absence of sexual attraction, other factors may drive partner selection (Edge & Vonk, 2024; Scheller et al., 2023).

#### Relationship Interests and Partner Preferences

Importantly, a possible source of influence on which attributes one desires in a partner is what relationship(s) one pursues. The overall lower preference ratings of asexual women suggest that a greater disinterest in long-term relationships may at least partially explain this. However, controlling for disinterest in long-term relationships, the effects did not disappear but became more evident. Interestingly, effects for financially secure-successful and confident-assertive even became substantial. Disinterest in long-term relationships does not appear to explain asexual women’s tendency of placing less importance on almost all preference dimensions.

Additionally, controlling for interest in purely sexual relationships and parenting interest revealed that differences between asexual and heterosexual women in partner preferences hold; however, depending on which relationship interest is controlled for, certain effects change markedly in size. For example, the substantial group difference for preference in a sexually experienced partner decreased noticeably; thereby rendering the effect inconclusive on whether it can be considered substantial, when controlling for interest in purely sexual relationships. Sexual experience also seems to be less important for heterosexual women when they are not pursuing purely sexual encounters. An alternative interpretation would be that through the matching on interest in sexual, non-romantic relationships, the more “typical” heterosexual women were filtered out, attenuating the previously observed differences. It was noteworthy that the effect for financially secure-successful did increase when controlling for parenting interest via matching, as compared to the main analysis. Given that heterosexual women are more interested in being parents, this result is not intuitive and awaits further qualification in future research.

To conclude, relationship interests do affect preferences for an ideal long-term partner, but differences in relationship interests do not account for overall lower preference ratings of asexual women. Exploring how these overall lower preference ratings of asexual women can be explained poses an interesting endeavor for future research.

#### Relationship Interests, Partner Preferences, and Romantic Orientation

Importantly, the study did not control for the romantic orientation of participants. Whether a person identifies as aromantic or romantic is likely to influence what and whether a relationship is desired and what attributes one seeks in a partner. Carvalho and Rodrigues (2022) showed that aromantic asexual individuals compared to romantic asexual individuals were more averse to sex, had less sexual experience, were less interested in romantic relationships, and identified more with asexuality. Further, findings by Scheller et al. (2023) suggest that sexual and romantic attraction differentially moderate sex differences in partner preferences. Future studies should include an item asking about romantic orientation besides sexual orientation to get a more differentiated picture of asexual interests and preferences.

### Personality

Asexual women consistently rated themselves significantly lower on all characteristics than their heterosexual counterparts. Asexual women rated themselves lower on confidence and assertiveness (H3.1). While this difference is likely to be explained by unobserved confounding, it is in line with asexual individuals having been shown to be less extraverted (Bogaert et al., 2018; Greaves et al., 2021), more socially withdrawn (Brotto et al., 2010), and showing less approach motivation in relationships (Greaves et al., 2021). This suggests that asexual individuals may engage less in socializing than heterosexual individuals.

Consistent with the finding that asexual women have a more negative body image (Swami et al., 2019), asexual women were found to rate themselves as less attractive (H3.2). Perhaps being confronted with heteronormative expectations as well as possible bias and discrimination negatively influences the self-perception of asexual individuals leading to more negative evaluations of their appearance. It is also possible that other variables mediate this relationship between asexuality and self-perceived attractiveness such as higher levels of honesty-humility that have been found in asexual individuals compared to heterosexual individuals (Greaves et al., 2021). Perhaps asexual individuals are more modest—a facet of honesty-humility—in their self-descriptions than heterosexual individuals. Asexual women were less sexually experienced than heterosexual women (H3.3) and this difference is unlikely to be explained by unobserved confounding. This result is in accordance with expectations and previous research (Aicken et al., 2013; Bogaert, 2004).

We also found some differences in further exploratory analyses. Asexual women scored lower on self-rated financial security and successfulness. As our sensitivity analysis suggests, this difference is likely to be explained by unobserved confounding. For instance, it could be that the difference can be explained by level of education. Consistent with this possibility, Bogaert (2004) found that asexual individuals were less educated than allosexual individuals. In contrast, however, other research (Antonsen et al., 2020; Greaves et al., 2017) did not find such an association. Education level was not directly measured in the current study. However, there was a small but inconclusive difference for self-perceived education and intelligence. Asexual women rated themselves lower on this dimension. It could be interesting for future studies to explore how objective levels of education and intelligence measures link to subjective self-ratings and whether this link is different for asexual individuals compared to heterosexual individuals. Exploratory analysis also showed that asexual women rated themselves lower in kindness and supportiveness, but evidence whether this effect is substantial or not remains inconclusive.

### Self-Ratings and Ideal Partner Preferences

The pattern of consistently lower self-ratings of asexual women together with the (mostly) lower importance ratings for partner preferences aligns with the similarity attraction hypothesis (see Dijkstra & Barelds, 2008). Self-perceived mate value describes how individuals assess their own characteristics in terms of being able to attract a partner (Fisher et al., 2008), and previous studies have shown lower self-perceived mate value to go along with lower ideal standards (Charlot et al., 2020). Some measures of self-perceived mate value such as the *Mate Value Inventory* (Kirsner et al., 2003) and the measure used by Charlot et al. (2020) closely resemble the self-rating measure used in this study. Thus, while not directly tested within the current study, it may be speculated that asexual and heterosexual women seek out partners that are more like themselves in terms of overall (self-perceived) desirability.

### Heterogeneity Among Asexual Individuals

Importantly, previous research has found that asexual individuals are very heterogeneous regarding their interests and preferences (e.g., Dawson et al., 2016; Hille, 2023). This study corroborates this heterogeneity, but highlights that there do seem to be distinct differences between asexual and heterosexual individuals. As noted earlier, diversity among asexual individuals may be caused by differences in romantic tendencies. For example, romantic asexual individuals are more likely to be in or want a romantic relationship than aromantic asexual individuals (Carvalho & Rodrigues, 2022). Likewise, Copulsky and Hammack (2023) found differences in sexual desire and behavior between graysexual, demisexual and asexual individuals. For example, asexual individuals were least likely to report romantic attraction and to be in a relationship. These nuances of asexuality have not been measured within this study but are likely to affect relationship interests and partner preferences. Future studies should examine how these different asexual identities and their intersection influence preferences and interests for intimate relationships.

### Limitations

The current study is not without limitations. As mentioned above, romantic orientation was not measured within this study. It is likely that whether a person identifies as romantic or aromantic has an influence on the relationship that a person pursues, or which attributes are endorsed in a potential partner (Carvalho & Rodrigues, 2022; Scheller et al., 2023).

Another limitation is that asexuality was self-assessed, and no extended definition of asexuality was provided when participants were asked about their sexual orientation. Thus, it is possible that participants have different understandings of what asexuality means (e.g., whether it also includes identities such as demisexual or graysexual). However, asexual women had less lifetime sexual partners, described themselves as significantly less sexually experienced, and were significantly less interested in a purely sexual relationship, indicating that asexual individuals were in fact successfully classified.

As a caveat, the sample mostly consisted of women and even though results did not change when including men and genderqueer/non-binary individuals, they were still underrepresented in the sample. Relatedly, it was not possible to find a matching “twin” with the same gender for each genderqueer/nonbinary asexual person. Hence, results are limited to asexual women and generalizations to other genders should only be made very cautiously. The inclusion of a sample with asexual and heterosexual men would enable testing whether sex differences in mate preferences typically found in heterosexual samples—such that men value physical attractiveness more than women and women value financial prospects and resources more so than men (Walter et al., 2020)—are less pronounced when sexual attraction is absent or reduced (see also Scheller et al., 2023). Considering different relationship types, one could expect deviations from patterns of sex differences in interest in purely sexual or romantic relationships. That is, while heterosexual men may prefer sexual, non-romantic relationships more so than heterosexual women, no such pattern might be observable when comparing asexual men and women. Similarly, we may expect heterosexual women to endorse romantic, non-sexual relationships more so than heterosexual men but might see different patterns among asexual individuals.

In addition, participants came from 38 different countries, but the majority were from Western countries such as the U.S., Germany, and Canada. Future research should focus on investigating diverse samples that are more representative of non-Western cultures. Further, in our study, no information was available for ethnicity, socioeconomic status, and education level. Findings from a study using a UK probability sample suggested that asexualindividuals, compared to heterosexual individuals, might be less educated, from a lower socioeconomic background, and less likely to be Caucasian (Bogaert, 2004). However, other studies found no evidence for differences in education level or socioeconomic status between asexual and allosexual individuals (Antonsen et al., 2020; Greaves et al., 2017; Prause & Graham, 2007). Including such demographics in future studies will be vital to resolve those apparent discrepancies and further, to rule out potential confounding by these variables. Moreover, for some effect estimates no definite conclusion could be reached about whether they can be considered substantial or not. Larger sample sizes are needed to determine whether those inconclusive effects are substantial or not.

### Conclusion

The present study is one of the first to provide evidence for differences between asexual and heterosexual women in their preferred relationship options, partner preferences, and personality. Although heterogeneity is high among asexual women, they were found to be distinctly different from heterosexual women. The current findings provide a solid basis for future research to build upon and can be used as a stepping stone when further investigating differences between asexual individuals and individuals from other sexual orientations to examine the full spectrum of human sexuality.

## Supplementary Information

Below is the link to the electronic supplementary material.

Supplementary file1 (DOCX 2862 KB)

## Data Availability

This study is part of a larger project, the Ideal Partner Survey, for which supporting information including the overarching project preregistration can be found on the Open Science Framework (OSF): https://osf.io/m4c8f/. The present study was not formally preregistered but an internal preregistration was submitted on March 1, 2022, as part of a Master thesis course at the University of Göttingen. This internal preregistration is available on the OSF (https://osf.io/ytcze). Data from the Ideal Partner Survey has been used in two other published studies before (Botzet et al., 2023; Kuschel et al., 2025). Thus, Laura J. Botzet, Amanda Shea, Virginia J. Vitzthum, and Tanja M. Gerlach had access to the data prior to the preregistration of the current project. However, in all past analyses asexual individuals were excluded and therefore nothing was known about this subgroup prior to data analysis. Paula C. Bange did not have access to the data until after submission of the internal preregistration. Due to the sensitive nature of the data, it is not possible to make it publicly available. However, based on Quintana’s (2020) primer for synthetic datasets, we created a synthetic dataset that mimics many of the features of the real data such as means and bivariate associations. This synthetic dataset was uploaded to the Open Science Framework (https://osf.io/rfgj4/) and thus can be used by others to run the code pertaining to this project with a realistic fake dataset.

## Footnotes

1 While Bogaert et al. (2018) used the HEXACO Personality Inventory-Revised (Lee & Ashton, 2018) to assess personality, Greaves et al. (2021) employed the MINI-IPIP6 personality scale (Sibley et al., 2011). Although the scales show some differences in the organization of their subscales, both capture the six personality dimensions (hence the label Big Six) of openness, conscientiousness, extraversion, agreeableness, emotional stability, and honesty-humility.

2 The study by Hall and Knox (2022) in which U.S. college students self-identifying as asexual were found to have lower parenting desire than their allosexual counterparts was not yet published when we preregistered the hypotheses of the present study. The same is true for Edge and Vonk (2024) who found that asexual individuals have a lower desire for a partner who wants children. Thus, we did not formulate an a-priori, directional hypothesis for wanting to become a parent.

3 Included men were mostly from the U.S. (71%) and on average 32.39 years old (*SD* = 12.26, ranging from 18 to 85 years). Nonbinary/genderqueer participants were also mostly from the U.S. (37%) and had a mean age of 23.80 years (*SD* = 5.60, ranging from 18 to 43 years).

4 Gender was used as a covariate for the robustness check (including men and genderqueer/nonbinary individuals besides women).

5 For a detailed comparison of balance properties across the different matching algorithms, see Table S5 in the supplemental material.

6 For additional information on the propensity score matching procedure, see supplement S3.

7 It is possible that individuals who identify as a sub-identity of the asexuality spectrum such as demisexual or graysexual selected the Other option instead of the Asexual option. However, as the answer option did not include a possibility to specify this, we do not know whether this is the case and consequently excluded all participants that chose this option.

8 For a detailed description of the classifying scheme, see the supplement S9.

9 Women and genderqueer/nonbinary individuals were among the unbalanced covariates. Thirty out of the 460 matched pairs (7%) did not have the same gender. Of these, 28 were asexual genderqueer/nonbinary individuals that were matched with heterosexual women. One asexual man was matched with a heterosexual woman and one asexual genderqueer/nonbinary person was matched with a heterosexual man.

10 In addition to Rosenbaum’s sensitivity analysis, we also calculated E-values (VanderWeele & Ding, 2017). See section S21 in the supplement for details.

11 Intercorrelations between outcomes split by outcome section are reported in supplement S10–S12.

12 In the partner preference sample, asexual women had on average 1.87 lifetime sexual partners (*SD* = 4.83) compared to 4.46 sexual partners (*SD* = 8.93) for heterosexuals. Lifetime number of sexual partners was significantly correlated with preference for a sexually experienced partner (*r* = .26, CI [.20, .33]).

13 Values are based on the self-rating sample (*n* = 386 asexual and matched heterosexual individuals, *n* = 45,058 for the unmatched heterosexual individuals).

## Code Availability

All code (for data wrangling and statistical analyses) is accessible via the corresponding project on the Open Science Framework (https://osf.io/y39u7/) and on GitHub (https://github.com/paulabange/idealpartnersurvey_asexuality_psm).
