# Supplementary material for: What Do Asexual Women Want? A Propensity Score Matching Study of Preferred Relationship Options and Ideal Partner Preferences
Source: Arch Sex Behav. 2026 Feb 5;55(1):345–68. doi: 10.1007/s10508-025-03365-2 (PMC12917066; doi:10.1007/s10508-025-03365-2)
Supplement: Supplementary file 1 — Supplementary file1 (DOCX 2862 KB) [file 10508_2025_3365_MOESM1_ESM.docx]

# Supplemental Material

**What Do Asexual Women Want? A Propensity Score Matching Study of Preferred Relationship Options and Ideal Partner Preferences**

Paula C. Bange^1,2^, Laura J. Botzet^2,3^, Amanda A. Shea^4^, Virginia J. Vitzthum^5,6^, & Tanja M. Gerlach^7,8^

^1^ Department of Developmental Psychology, Tilburg University, Tilburg, The Netherlands

^2^ Biological Personality Psychology, University of Goettingen, Goettingen, Germany

^3^ Leibniz ScienceCampus Primate Cognition, Goettingen, Germany

^4^ Clue by BioWink GmbH, Berlin, Germany

^5^ CEMCOR, Department of Medicine, University of British Columbia, Vancouver, British Columbia, Canada

^6^ Department of Anthropology, Indiana University, Bloomington, Indiana, USA

^7^ Leibniz Institute for Educational Trajectories (LIfBi), Bamberg, Germany

^8^ School of Psychology, Queen's University Belfast, Belfast, United Kingdom

#

## Table of Contents

[S1](#_184mhaj) Summary of Hypotheses

[S2](#_184mhaj) Flowchart of Applied Exclusion Criteria

[S3 Additional Information on Propensity Score Matching Procedure](#_184mhaj)

[S4](#_3s49zyc) Distributions of Propensity Scores in the Matched and Unmatched Asexual and Heterosexual Samples

[S5 Comparison of Balance Properties Before and After Matching Across all Matching Algorithms](#_3s49zyc)

[S6](#_3s49zyc) Means, Standardized Mean Differences, and Variance Ratios Before and After Matching

[S7 Comparison of Asexual Individuals, Matched Heterosexual Individuals, and Unmatched Heterosexual Individuals on all Covariates and Outcomes](#_279ka65)

[S8 Complete List of all Countries With Participant Counts and Percentages](#_36ei31r)

[S9 Measures and Indices: Classifying Scheme for Relationship Status](#_1ljsd9k)

[S10 Zero-Order Correlations Between all Preferred Relationship Option Variables](#_45jfvxd)

[S11 Zero-Order Correlations Between all Ideal Partner Preference Variables](#_2koq656)

[S12 Zero-Order Correlations Between all Self-Rating Variables](#_zu0gcz)

[S13 Testing Differences Between Asexual and Heterosexual Women in Ideal Partner Preferences and Self-Ratings on Item-Level](#_3jtnz0s)

[S14 Results for Additional Analyses Controlling for Influence of Relationship Interests on Partner Preferences](#_1yyy98l)

[S15 Effect Estimates and 95% Confidence Intervals for Additional Analyses Controlling for Relationship Interests](#_4iylrwe)

[S16 Comparison of Balance Properties Before and After Matching for the Robustness Check](#_2y3w247)

[S17 Love Plot Displaying Standardized Mean Differences Before and After Matching of all Covariates for the Robustness Check](#_3x8tuzt)

[S18 Distributions of Propensity Scores in the Matched and Unmatched Asexual and Heterosexual Samples for the Robustness Analysis](#_2ce457m)

[S19 Results of t-Tests for the Robustness Check](#_rjefff)

[S20 Effect Sizes and 95% Confidence Intervals for the Robustness Analyses](#_3bj1y38)

[S21 Additional Sensitivity Analysis Using E-values](#_ft7jo0zp3mz)

## S1 Summary of Hypotheses

**Table S1**

*Summary of all Hypotheses*

| Outcome | Hypothesis wording |
| --- | --- |
| Preferred relationship options |  |
| H1.1.  Sexual, non-romantic relationship(s) | Asexual women show *lower* interest in having sexual, non-romantic relationships than heterosexual women. |
| H1.2.  Non-sexual, romantic relationship(s) | Asexual women show *higher* interest in having non-sexual, romantic relationships than heterosexual women. |
| H1.3.  Non-monogamous relationship(s) | Asexual women show *higher* interest in having non-monogamous relationships than heterosexual women. |
| H1.4.  Alternative committed relationship(s) | Asexual women show *higher* interest in having alternative committed relationships than heterosexual women. |
| H1.5.  Being non-partnered | Asexual women show *higher* interest in being non-partnered than heterosexual women. |
| Ideal partner preferences |  |
| H2.1.  Confidence and assertiveness | Asexual women show *lower* importance ratings for a confident and assertive long-term partner than heterosexual women. |
| H2.2.  Attractiveness | Asexual women show *lower* importance ratings for an attractive long-term partner than heterosexual women. |
| H2.3.  Sexual experience | Asexual women show *lower* importance ratings for a sexually experienced long-term partner than heterosexual women. |
| Self-ratings |  |
| H3.1.  confidence and assertiveness | Asexual women rate themselves *lower* in confidence and assertiveness than heterosexual women. |
| H3.2.  Attractiveness | Asexual women rate themselves *lower* in attractiveness than heterosexual women. |
| H3.3.  Sexual experience | Asexual women rate themselves *lower* in sexual experience than heterosexual women. |

*Note.* The enumeration of the hypotheses was based on the internal preregistration.

## S2 Flowchart of Applied Exclusion Criteria

**Figure S1**

*Flowchart of Applied Exclusion Criteria*

**
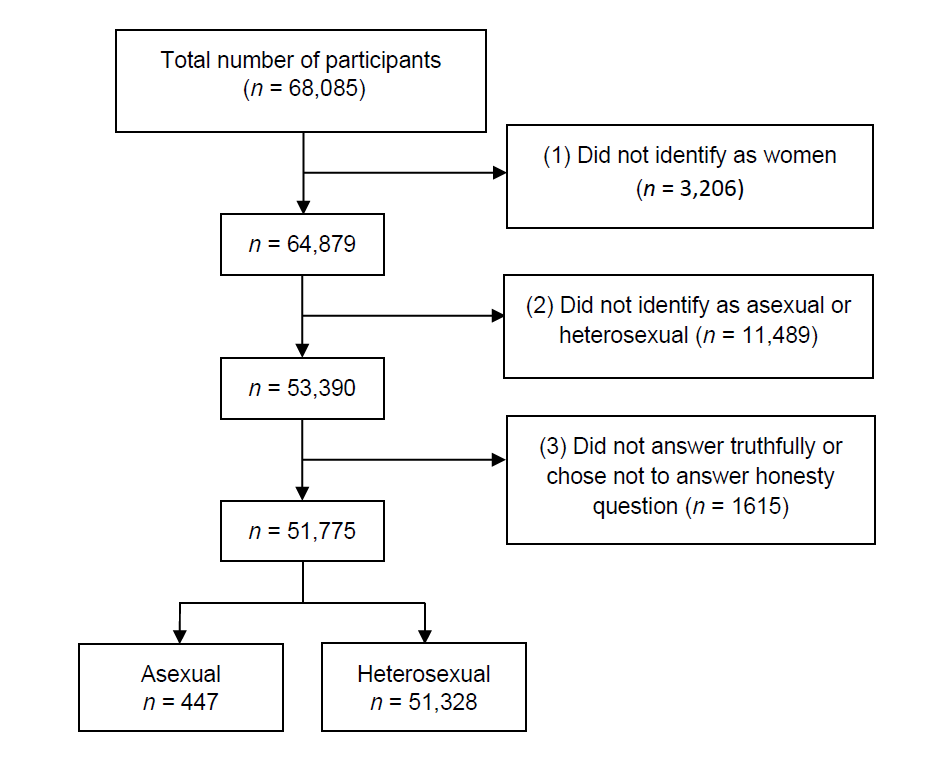
**

*Note.* The total number of participants refers to all participants indicating they were 18 years or older. Participation in the survey was not possible, if participants indicated being younger than 18.

## S3 Additional Information on Propensity Score Matching Procedure

Propensity score matching cannot handle missing values; therefore prior to calculating the propensity score, all observations that included missings were thrown out. Moreover, for the matching process, categorical variables with more than two categories are transformed into several binary variables. Each category then has its own binary indicator variable. To illustrate, for the country variable there were over 100 different categories (or countries). For example, being from the country Italy was then coded into 0 = *not from Italy* and 1 = *from Italy*. This was done for all possible categories/countries respectively. Participants were then matched based on these binary indicator variables.

When matching with the nearest-neighbor-matching, it is possible to limit the distance between propensity scores of two participants that are to be matched. Caliper width is measured in standard deviations of the logit of the propensity score. A tight caliper can be useful when balance after matching is poor. However, this can lead to a lower number of matched pairs as participants may be discarded in the process.

## S4 Distributions of Propensity Scores in the Matched and Unmatched Asexual and Heterosexual Samples

**Figure S4**

*Distributions of Propensity Scores in the Matched and Unmatched Asexual and Heterosexual Samples*

*
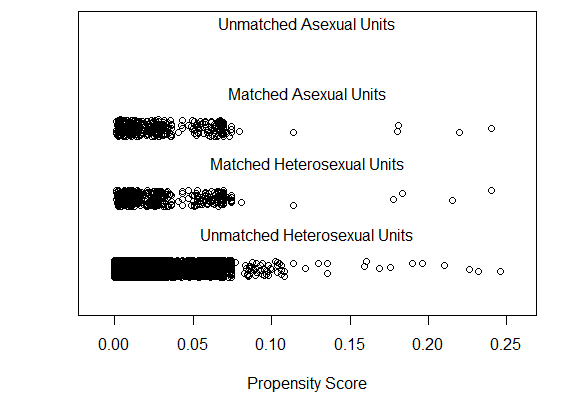
*

*Note.* Represented are the distributions for the partner preference analysis sample. There are no unmatched asexual units displayed because no asexual participant was discarded during the matching procedure.

## S5 Comparison of Balance Properties Before and After Matching Across all Matching Algorithms

**Table S5**

*Balance Properties Before Matching and After Matching for all Matching Algorithms*

| Covariates | Before matching  (*N* = 47,136) | | Optimal matching  (*N* = 780) | | Nearest-neighbor-  matching  (*N* = 780) | | Nearest-neighbor-  matching with  caliper = 0.25  (*N* = 778) | | Nearest-neighbor-  matching with  caliper = 0.10  (*N* = 774) | |
| --- | --- | --- | --- | --- | --- | --- | --- | --- | --- | --- |
|  | *SMD* | *VR* | *SMD* | *VR* | *SMD* | *VR* | *SMD* | *VR* | *SMD* | *VR* |
| Propensity score | 0.75 | 4.82 | 0.00 | 1.01 | 0.00 | 1.00 | 0.00 | 1.00 | 0.00 | 1.00 |
| Language |  |  |  |  |  |  |  |  |  |  |
| - English | 0.54 | - | 0.39 | - | 0.01 | - | 0.01 | - | 0.01 | - |
| - German | 0.04 | - | −1.01 | - | 0.00 | - | 0.00 | - | 0.00 | - |
| - Danish | −0.11 | - | 0.12 | - | 0.00 | - | 0.00 | - | 0.00 | - |
| - French | −0.54 | - | 0.25 | - | −0.01 | - | −0.01 | - | −0.01 | - |
| - Japanese | 0.04 | - | 0.18 | - | 0.00 | - | 0.00 | - | 0.00 | - |
| - Chinese | −0.08 | - | 0.00 | - | 0.00 | - | 0.00 | - | 0.00 | - |
| - Portuguese | −0.11 | - | 0.23 | - | 0.00 | - | 0.00 | - | 0.00 | - |
| - Spanish | −0.28 | - | −0.12 | - | 0.00 | - | 0.00 | - | 0.00 | - |
| - Russian | 0.01 | - | 0.12 | - | 0.00 | - | 0.00 | - | 0.00 | - |
| - Italian | −0.13 | - | 0.20 | - | −0.01 | - | −0.01 | - | 0.00 | - |
| Age | −0.20 | 0.77 | −0.35 | 0.54 | −0.03 | 0.94 | −0.03 | 0.94 | −0.02 | 0.96 |
| Relationship status |  |  |  |  |  |  |  |  |  |  |
| - Long-term relationship | 0.99 | - | 0.09 | - | 0.02 | - | 0.02 | - | 0.02 | - |
| - Dating/Sexual relationship | −0.37 | - | 0.02 | - | −0.01 | - | −0.01 | - | −0.01 | - |
| - No relationship | −0.86 | - | −0.09 | - | −0.02 | **-** | −0.02 | - | −0.02 | - |
| Relationship length | −0.35 | 0.58 | −0.02 | 0.66 | −0.01 | 0.96 | −0.01 | 0.96 | −0.01 | 0.96 |
| Discarded^a^ | - | | 0 | | 0 | | 1 | | 3 | |
| Unbalanced^b^ | - | | 27 | | 0 | | 0 | | 0 | |

*Note. SMD* = Standardized mean difference. *VR* = Variance ratio. Values are based on the propensity-score matched sample for the partner preference analysis. Sample sizes are given in brackets. Country is not included in the table because of space restrictions. Nearest-neighbor-matching algorithms were run without replacement.

^a^ Discarded refers to the number of asexual participants that could not be matched during the matching process.

^b^ Unbalanced refers to the number of covariates that could not be balanced through matching (including the country variables).

## S6 Means, Standardized Mean Differences, and Variance Ratios Before and After Matching

**Table S6**

*Means, Standardized Mean Differences, and Variance Ratios Before and After Matching*

| Covariates | Before Matching | | | After Matching | | |
| --- | --- | --- | --- | --- | --- | --- |
|  | Asexual  Individuals | Heterosexual  Individuals | *SMD (VR)* | Asexual  Individuals | Heterosexual Individuals | *SMD (VR)* |
|  | *M* | *M* |  | *M* | *M* |  |
| Propensity score | 0.03 | 0.01 | 0.75 (4.82) | 0.03 | 0.03 | 0.00 (1.00) |
| Language |  |  |  |  |  |  |
| English | 0.50 | 0.23 | 0.54 | 0.50 | 0.49 | 0.01 |
| German | 0.15 | 0.14 | 0.04 | 0.15 | 0.15 | 0.00 |
| Danish | 0.02 | 0.03 | −0.11 | 0.02 | 0.02 | 0.00 |
| French | 0.06 | 0.20 | −0.54 | 0.06 | 0.07 | −0.01 |
| Japanese | 0.03 | 0.02 | 0.04 | 0.03 | 0.03 | 0.00 |
| Chinese | 0.00 | 0.01 | −0.08 | 0.00 | 0.00 | 0.00 |
| Portuguese | 0.05 | 0.07 | −0.11 | 0.05 | 0.05 | 0.00 |
| Spanish | 0.13 | 0.23 | −0.28 | 0.13 | 0.13 | 0.00 |
| Russian | 0.02 | 0.02 | 0.01 | 0.02 | 0.02 | 0.00 |
| Italian | 0.04 | 0.06 | −0.13 | 0.04 | 0.04 | −0.01 |
| Age | 23.94 | 25.20 | −0.20 (0.77) | 23.94 | 24.12 | −0.03 (0.94) |
| Relationship status |  |  |  |  |  |  |
| No relationship | 0.69 | 0.23 | 0.99 | 0.69 | 0.70 | 0.02 |
| Dating/Sexual relationship | 0.05 | 0.14 | −0.37 | 0.05 | 0.06 | −0.01 |
| Long-term relationship | 0.26 | 0.63 | −0.86 | 0.26 | 0.25 | −0.01 |
| Relationship length (in years) | 1.16 | 2.32 | −0.35 (0.58) | 1.16 | 1.18 | −0.01 (0.96) |

*Note. SMD* = standardized mean difference. *VR* = variance ratio. Variance ratios can only be computed for continuous variables. Matching was done using the nearest-neighbor algorithm. Country variables are not displayed in the table due to space restrictions. Sample after matching based on matched sample for partner preferences (*N* = 780).

## S7 Comparison of Asexual Individuals, Matched Heterosexual Individuals, and Unmatched Heterosexual Individuals on all Covariates and Outcomes

**Table S7**

*Comparison of Asexual Individuals, Matched Heterosexual Individuals, and Unmatched Heterosexual Individuals on all Covariates and Outcomes*

| Variables | Asexual v. Matched heterosexual individuals  (Main analysis) | | | | | Asexual v. Unmatched heterosexual  individuals | | | | | Matched heterosexual v. Unmatched heterosexual individuals | | | | |
| --- | --- | --- | --- | --- | --- | --- | --- | --- | --- | --- | --- | --- | --- | --- | --- |
|  | *t*/*Χ*^2^ | *df* | *p* | *d*/*V* | 95% CI | *t*/*Χ*^2^ | *df* | *p* | *d*/*V* | 95% CI | *t*/*Χ^2^* | *df* | *p* | *d*/*V* | 95% CI |
| Covariates^a^ |  |  |  |  |  |  |  |  |  |  |  |  |  |  |  |
| Age | −0.86 | 389 | .39 | −0.03 | [−0.09, 0.03] | −3.89 | 397.57 | <.001 | −0.17 | [−0.27, −0.07] | −3.24 | 397.03 | .001 | −0.15 | [−0.25, −0.05] |
| Country | 9.98 | 37 | 1 | 0.11 | [0.00, 1.00] | 299.08 | 169 | <.001 | 0.08 | [0.07, 0.09] | 324.44 | 169 | <.001 | 0.08 | [0.08, 0.09] |
| Language | 0.06 | 8 | 1 | 0.01 | [0.05, 0.15] | 189.13 | 9 | <.001 | 0.06 | [0.05, 0.08] | 182.37 | 9 | <.001 | 0.06 | [0.05, 0.07] |
| Relationship status | 0.12 | 2 | .94 | 0.01 | [0.01, 0.10] | 462.21 | 2 | <.001 | 0.10 | [0.09, 0.11] | 477.66 | 2 | <.001 | 0.10 | [0.09, 0.11] |
| Relationship length (in years) | −0.12 | 389 | .91 | −0.01 | [−0.15, 0.13] | −6.95 | 400.32 | <.001 | −0.27 | [−0.37, −0.17] | −6.66 | 399.90 | <.001 | −0.26 | [−0.36, −0.16] |
| Preferred relationship options (*N* =780) |  | 322 |  |  |  |  |  |  |  |  |  |  |  |  |  |
| Sexual, non-romantic relationship | −8.96 |  | <.001 | −0.67 | [−0.83, −0.51] | −11.49 | 332.15 | <.001 | −0.67 | [−0.83, −0.52] | 2.09 | 327.34 | .0370 | −0.67 | [−0.83, −0.51] |
| Non-sexual, romantic relationship | 14.44 |  | <.001 | 1.12 | [0.93, 1.32] | 24.71 | 328.96 | <.001 | 1.24 | [1.13, 1.35] | 3.54 | 327.97 | .0005 | 0.19 | [0.08, 0.30] |
| Monogamous relationship | −6.11 |  | <.001 | −0.46 | [−0.83, −0.51] | −5.83 | 328.01 | <.001 | −0.31 | [−0.42, −0.20] | 2.21 | 329.97 | .0279 | 0.10 | [−0.01, 0.21] |
| Non-monogamous relationship | 2.58 |  | .01 | 0.20 | [0.05, 0.35] | 2.82 | 327.18 | .0050 | 0.16 | [0.05, 0.27] | −0.61 | 328.27 | .5451 | −0.03 | [−0.14, 0.08] |
| Alternative committed relationship | 7.58 |  | <.001 | 0.57 | [0.41, 0.73] | 15.35 | 331.65 | <.001 | 0.66 | [0.55, 0.77] | 2.95 | 327.95 | .0034 | 0.16 | [0.05, 0.27] |
| Being non-partnered | 8.27 |  | <.001 | 0.61 | [0.45, 0.76] | 14.45 | 326.70 | <.001 | 0.88 | [0.77, 0.99] | 4.81 | 328.09 | <.001 | 0.26 | [0.15, 0.37] |
| Becoming a parent | −10.29 |  | <.001 | −0.77 | [−0.94, −0.64] | −12.41 | 327.31 | <.001 | −0.71 | [−0.82, −0.60] | 1.12 | 327.91 | .2638 | 0.06 | [−0.05, 0.17] |
| Ideal partner preferences (*N* = 772) |  | 389 |  |  |  |  |  |  |  |  |  |  |  |  |  |
| Confident-assertive | −5.75 |  | <.001 | −0.40 | [−0.51, −0.24] | −8.79 | 393.49 | <.001 | −0.54 | [−0.64, −0.44] | −8.79 | 393.49 | <.001 | −0.54 | [−0.64, −0.44] |
| Attractive | −7.32 |  | <.001 | −0.52 | [−0.66, −0.37] | −9.31 | 392.90 | <.001 | −0.61 | [−0.71, −0.51] | −9.31 | 392.9 | <.001 | −0.61 | [−0.71, −0.51] |
| Sexually experienced | −9.73 |  | <.001 | −0.66 | [−0.81, −0.51] | −17.09 | 396.16 | <.001 | −0.83 | [−0.93, −0.73] | −17.10 | 396.16 | <.001 | −0.83 | [−0.93, −0.73] |
| Kind-supportive | -2.28 |  | .02 | -0.16 | [-0.30, -0.02] | -0.85 | 394.10 | .40 | -0.05 | [-0.15, 0.05] | -0.85 | 394.10 | .40 | -0.05 | [-0.15, 0.05] |
| Financially secure/successful | -3.06 |  | .00 | -0.21 | [-0.35, -0.08] | -4.05 | 394.44 | <.001 | -0.23 | [-0.33, -0.13] | -4.05 | 394.44 | <.001 | -0.23 | [-0.33, -0.17] |
| Educated-intelligent | 0.21 |  | .83 | 0.01 | [-0.12, 0.15] | -0.17 | 394.94 | .87 | -0.01 | [-0.11, 0.09] | -0.17 | 394.94 | .87 | -0.01 | [-0.11, 0.09] |
| Self-ratings (*N* = 646) |  | 385 |  |  |  |  |  |  |  |  |  |  |  |  |  |
| Confident-assertive | -6.30 |  | <.001 | -0.43 | [-0.57, -0.29] | -10.56 | 389.91 | <.001 | -0.63 | [-0.73, -0.53] | -3.00 | 391.44 | .00 | -0.16 | [-0.26, -0.06] |
| Attractive | -7.00 |  | <.001 | -0.49 | [-0.63, -0.34] | -11.33 | 389.97 | <.001 | -0.67 | [-0.77, -0.57] | -2.71 | 391.45 | .01 | -0.14 | [-0.24, -0.04] |
| Sexually experienced | -10.72 |  | <.001 | -0.65 | [-0.79, -0.52] | -25.91 | 394.53 | <.001 | -1.11 | [-1.21, -1.01] | -8.96 | 390.96 | <.001 | -0.48 | [-0.58, -0.38] |
| Kind-supportive | -5.70 |  | <.001 | -0.40 | [-0.54, -0.25] | -6.74 | 389.70 | <.001 | -0.41 | [-0.51, -0.31] | 0.36 | 392.31 | .72 | -0.02 | [-0.08, 0.12] |
| Financially secure/successful | -6.45 |  | <.001 | -0.45 | [-0.60, -0.31] | -9.16 | 390.58 | <.001 | -0.51 | [-0.61, -0.41] | -0.70 | 391.70 | .48 | -0.04 | [-0.14, 0.06] |
| Edcuated-intelligent | -2.54 |  | .01 | -0.17 | [-0.31, -0.04] | -2.83 | 391.03 | .01 | -0.15 | [-0.25, -0.05] | 0.61 | 390.82 | .54 | 0.03 | [-0.07, 0.13] |

*Note. d* = Cohen’s d. *V* = Cramer’s V. CI = confidence interval; For each outcome section, values are based on the respective propensity-score matched sample for that outcome section. For binary variables a chi square test was computed. For continuous variables a t-test was calculated. Note that for the covariate country, assumptions for the chi square test were not met. More than 20% of cells contained less than five observations. Instead, a Fisher’s exact test should have been reported. However, to give an effect estimate and a corresponding confidence interval, chi square tests were reported. See the code on the OSF project side ([https://osf.io/m4c8f](https://osf.io/m4c8f/)) for Fisher’s exact tests.

^a^ Values for covariates are based on the partner preference sample. All other values are based on the respective samples for the specified outcome section. Language and country variables are not presented in the table due to space restrictions.

## S8 Complete List of all Countries With Participant Counts and Percentages

**Table S8**

*Counts and Percentages of Participants for Each Country in Decreasing Order*

| Country | Count | % |
| --- | --- | --- |
| United States of America | 181 | 28.02 |
| Germany | 82 | 12.69 |
| Canada | 42 | 6.50 |
| United Kingdom | 42 | 6.50 |
| Brazil | 34 | 5.26 |
| Italy | 33 | 5.11 |
| France | 28 | 4.33 |
| Mexico | 28 | 4.33 |
| Australia | 22 | 3.41 |
| Japan | 20 | 3.10 |
| Spain | 18 | 2.79 |
| Denmark | 16 | 2.48 |
| Switzerland | 14 | 2.17 |
| Argentina | 13 | 2.01 |
| Russia | 12 | 1.86 |
| Colombia | 8 | 1.24 |
| Czechia | 7 | 1.08 |
| Austria | 6 | 0.93 |
| Chile | 6 | 0.93 |
| New Zealand | 6 | 0.93 |
| Finland | 3 | 0.46 |
| Guatemala | 3 | 0.46 |
| Panama | 3 | 0.46 |
| Philippines | 3 | 0.46 |
| Costa Rica | 2 | 0.31 |
| Netherlands | 2 | 0.31 |
| Singapore | 2 | 0.31 |
| South Africa | 2 | 0.31 |
| Sweden | 2 | 0.31 |
| Algeria | 1 | 0,15 |
| Dominican Republic | 1 | 0.15 |
| Iran | 1 | 0.15 |
| Ireland | 1 | 0.15 |
| Nicaragua | 1 | 0.15 |
| Pakistan | 1 | 0.15 |

*Note.* Due to rounding, percentages add up to 99.96% instead of 100%. Values are reported for the partner preference sample (*N* = 780).

## S9 Measures and Indices: Classifying Scheme for Relationship Status

For relationship status, three categories were formed: (1) long-term relationship, (2) dating/sexual relationship, and (3) no relationship. The answer options “*Long-term committed/exclusive sexual relationship with one or more partners*” and “*Ongoing (longer than 1 month) uncommitted/non-exclusive romantic and/or sexual relationship*” made up category (1) long-term relationships.

The answer options “*Short-term (casual) sexual relationship (e.g. hookups or one-night-stands)*” as well as “*New (less than 1 month old) romantic and/or sexual relationship*” made up category (2) dating/sexual relationship.

The answer option “*No romantic or sexual relationships during the past 3 months*” made up category (3) no relationship.

When only “*Other*” was selected as an answer, the value for relationship status was set to missing since no clear assignment to any of the categories was feasible.

Participants were able to select more than one relationship status. For participants that selected more than one status, I assigned each participant according to the following scheme to one of the three relationship categories: If one of the selected options included “*Long-term committed/exclusive sexual relationship with one or more partners*” or “*Ongoing (longer than 1 month) uncommitted/non-exclusive romantic and/or sexual relationship*” the participant was assigned to category (1) long-term relationship.

If none of the selected options included answers belonging to category (1) AND one of the selected answers is “*Short-term (casual) sexual relationship (e.g. hookups or one-night-stands)*” or “*New (less than 1 month old) romantic and/or sexual relationship*” the participant was assigned to category (2) dating/sexual relationship.

If none of the selected options included answers belonging to category (1) or category (2) AND “*No romantic or sexual relationships during the past 3 months”* was selected, participants were assigned to category (3) no relationship.

## S10 Zero-Order Correlations Between all Preferred Relationship Option Variables

**Table S10**

*Means, Standard Deviations, and Correlations With Confidence Intervals for Preferred Relationship Option Outcomes*

| Variable | *M* | *SD* | 1 | 2 | 3 | 4 | 5 | 6 |
| --- | --- | --- | --- | --- | --- | --- | --- | --- |
|  |  |  |  |  |  |  |  |  |
| 1. Sexual, non-romantic relationships | 1.19 | 1.68 |  |  |  |  |  |  |
|  |  |  |  |  |  |  |  |  |
| 2. Non-sexual, romantic relationships | 3.32 | 2.08 | -.14** |  |  |  |  |  |
|  |  |  | [-.21, -.06] |  |  |  |  |  |
|  |  |  |  |  |  |  |  |  |
| 3. Monogamous relationships | 4.68 | 1.62 | .02 | -.06 |  |  |  |  |
|  |  |  | [-.06, .09] | [-.14, .02] |  |  |  |  |
|  |  |  |  |  |  |  |  |  |
| 4. Non-monogamous relationships | 0.98 | 1.51 | .28** | .16** | -.19** |  |  |  |
|  |  |  | [.21, .35] | [.09, .24] | [-.27, -.12] |  |  |  |
|  |  |  |  |  |  |  |  |  |
| 5. Alternative committed relationships | 3.96 | 1.98 | .10* | .30** | -.08* | .20** |  |  |
|  |  |  | [.02, .18] | [.23, .37] | [-.16, -.00] | [.12, .27] |  |  |
|  |  |  |  |  |  |  |  |  |
| 6. Being non-partnered | 2.79 | 1.86 | .03 | .25** | -.29** | .12** | .25** |  |
|  |  |  | [-.05, .11] | [.17, .32] | [-.36, -.21] | [.05, .20] | [.17, .32] |  |
|  |  |  |  |  |  |  |  |  |
| 7. Becoming a parent | 3.17 | 2.28 | .08 | -.15** | .28** | -.08* | -.12** | -.36** |
|  |  |  | [-.00, .15] | [-.22, -.07] | [.21, .35] | [-.16, -.00] | [-.19, -.04] | [-.43, -.29] |
|  |  |  |  |  |  |  |  |  |

*Note.* Values in square brackets indicate the 95% confidence interval for each correlation. Values are based on the preferred relationship options analysis sample (*N* = 646).

* *p* < .05. ** *p* < .01.

## S11 Zero-Order Correlations Between all Ideal Partner Preference Variables

**Table S11**

*Means, Standard Deviations, and Correlations With Confidence Intervals for Partner Preference Outcomes*

| Variable | *M* | *SD* | 1 | 2 | 3 | 4 | 5 |
| --- | --- | --- | --- | --- | --- | --- | --- |
|  |  |  |  |  |  |  |  |
| 1. Confident-assertive | 4.17 | 1.08 |  |  |  |  |  |
|  |  |  |  |  |  |  |  |
| 2. Attractive | 3.50 | 1.40 | .27** |  |  |  |  |
|  |  |  | [.20, .33] |  |  |  |  |
|  |  |  |  |  |  |  |  |
| 3. Sexually experienced | 1.99 | 1.99 | .30** | .31** |  |  |  |
|  |  |  | [.24, .36] | [.24, .37] |  |  |  |
|  |  |  |  |  |  |  |  |
| 4. Kind-supportive | 5.49 | 0.64 | .11** | .05 | .00 |  |  |
|  |  |  | [.04, .18] | [-.02, .12] | [-.07, .07] |  |  |
|  |  |  |  |  |  |  |  |
| 5. Financially secure-successful | 4.24 | 1.12 | .39** | .28** | .23** | .10** |  |
|  |  |  | [.32, .44] | [.21, .34] | [.16, .29] | [.03, .17] |  |
|  |  |  |  |  |  |  |  |
| 6. Educated-intelligent | 4.90 | 1.03 | .32** | .27** | .16** | .17** | .37** |
|  |  |  | [.25, .38] | [.20, .33] | [.09, .23] | [.10, .24] | [.31, .43] |
|  |  |  |  |  |  |  |  |

*Note.* Values in square brackets indicate the 95% confidence interval for each correlation. Values are based on the partner preference analysis sample (*N* = 780).

* *p* < .05. ** *p* < .01.

## S12 Zero-Order Correlations Between all Self-Rating Variables

**Table S12**

*Means, Standard Deviations, and Correlations With Confidence Intervals for Self-Rating Outcomes*

| Variable | *M* | *SD* | 1 | 2 | 3 | 4 | 5 |
| --- | --- | --- | --- | --- | --- | --- | --- |
|  |  |  |  |  |  |  |  |
| 1. Confident-assertive | 3.51 | 1.26 |  |  |  |  |  |
|  |  |  |  |  |  |  |  |
| 2. Attractive | 3.33 | 1.24 | .49** |  |  |  |  |
|  |  |  | [.43, .54] |  |  |  |  |
|  |  |  |  |  |  |  |  |
| 3. Sexually experienced | 1.59 | 1.73 | .29** | .32** |  |  |  |
|  |  |  | [.22, .35] | [.25, .38] |  |  |  |
|  |  |  |  |  |  |  |  |
| 4. Kind-supportive | 4.85 | 0.90 | .20** | .26** | .22** |  |  |
|  |  |  | [.13, .27] | [.19, .32] | [.15, .28] |  |  |
|  |  |  |  |  |  |  |  |
| 5. Financially secure-successful | 3.64 | 1.14 | .46** | .43** | .24** | .26** |  |
|  |  |  | [.40, .51] | [.37, .49] | [.17, .30] | [.19, .32] |  |
|  |  |  |  |  |  |  |  |
| 6. Educated-intelligent | 4.54 | 0.90 | .41** | .39** | .21** | .27** | .54** |
|  |  |  | [.35, .47] | [.33, .45] | [.14, .28] | [.20, .33] | [.49, .59] |
|  |  |  |  |  |  |  |  |

*Note.* Values in square brackets indicate the 95% confidence interval for each correlation. Values are based on the self-rating sample (*N* = 772).

* *p* < .05. ** *p* < .01.

## S13 Testing Differences Between Asexual and Heterosexual Women in Ideal Partner Preferences and Self-Ratings on Item-Level

**Table S13**

*Results of Item-Level Analyses for Partner Preference and Self-Rating Dimensions*

| Outcome | Asexual  Women | | Heterosexual Women | | *df* | *t* | *p* | *d* | 95% CI |
| --- | --- | --- | --- | --- | --- | --- | --- | --- | --- |
|  | *M* | *SD* | *M* | *SD* |  |  |  |  |  |
| Ideal partner preferences |  |  |  |  |  |  |  |  |  |
| Confident-assertive | 3.97 | 1.15 | 4.37 | 0.97 | 389 | −5.75 | <.001 | −0.37 | [−0.51, −0.24] |
| - Confident | 4.22 | 1.42 | 4.69 | 1.18 | 389 | −5.54 | <.001 | −0.36 | [−0.49, −0.23] |
| - Assertive | 3.73 | 1.32 | 4.05 | 1.21 | 388 | −3.84 | <.001 | −0.26 | [−0.40, −0.12] |
|  |  |  |  |  |  |  |  |  |  |
| Attractive | 3.15 | 3.54 | 3.85 | 1.15 | 389 | −7.32 | <.001 | −0.52 | [−0.66, −0.37] |
| - Attractive body | 2.88 | 1.62 | 3.65 | 1.31 | 389 | −7.34 | <.001 | −0.53 | [−0.68, −0.38] |
| - Attractive face | 3.43 | 1.68 | 4.05 | 1.28 | 387 | −5.91 | <.001 | −0.42 | [−0.56, −0.27] |
|  |  |  |  |  |  |  |  |  |  |
| Sexually experienced | 1.37 | 1.85 | 2.62 | 1.93 | 389 | −9.73 | <.001 | −0.66 | [−0.81, −0.51] |
|  |  |  |  |  |  |  |  |  |  |
| Kind-supportive | 5.44 | 0.72 | 5.54 | 0.55 | 389 | −2.28 | .02 | −0.16 | [−0.30, −0.02] |
| - Kind | 5.48 | 0.90 | 5.66 | 0.62 | 388 | −3.18 | .00 | −0.23 | [−0.37, −0.08] |
| - Supportive | 5.40 | 0.84 | 5.43 | 0.78 | 382 | −0.53 | .60 | −0.04 | [−0.18, 0.10] |
|  |  |  |  |  |  |  |  |  |  |
| Financially secure-successful | 4.12 | 1.20 | 4.36 | 1.00 | 389 | −3.06 | .00 | −0.21 | [−0.35, −0.08] |
| - Financially secure | 4.23 | 1.43 | 4.28 | 1.36 | 387 | −0.45 | .65 | −0.03 | [−0.17, 0.10] |
| - Successful ambitious | 4.01 | 1.47 | 4.45 | 1.29 | 389 | −4.49 | <.001 | −0.31 | [−0.46, −0.17] |
|  |  |  |  |  |  |  |  |  |  |
| Educated-intelligent | 4.91 | 1.03 | 4.89 | 1.03 | 389 | 0.21 | .83 | 0.01 | [−0.12, 0.15] |
| - Educated | 4.76 | 1.85 | 4.76 | 1.93 | 388 | −0.03 | .98 | 0.00 | [−0.14, 0.14] |
| - Intelligent | 5.06 | 1.06 | 5.03 | 1.01 | 389 | 0.43 | .67 | 0.03 | [−0.11, 0.17] |
| Self-ratings |  |  |  |  |  |  |  |  |  |
| Confident-assertive | 3.24 | 1.31 | 3.77 | 1.15 | 385 | −6.30 | <.001 | −0.43 | [−0.57, −0.29] |
| - Confident | 3.17 | 1.46 | 3.32 | 1.36 | 384 | −6.19 | <.001 | −0.43 | [−0.57, −0.28] |
| - Assertive | 3.32 | 1.46 | 3.79 | 1.30 | 380 | −4.99 | <.001 | −0.34 | [−0.48, −0.20] |
|  |  |  |  |  |  |  |  |  |  |
| Attractive | 3.04 | 1.28 | 3.62 | 1.12 | 385 | −7.00 | <.001 | −0.49 | [−0.63, −0.34] |
| - Attractive body | 2.90 | 1.40 | 3.43 | 1.29 | 383 | −5.64 | <.001 | −0.40 | [−0.55, −0.26] |
| - Attractive face | 3.18 | 1.41 | 3.81 | 1.21 | 383 | −6.94 | <.001 | −0.47 | [−0.62, −0.33] |
|  |  |  |  |  |  |  |  |  |  |
| Sexually experienced | 1.05 | 1.44 | 2.13 | 1.82 | 385 | −10.72 | <.001 | −0.65 | [−0.79, −0.52] |
|  |  |  |  |  |  |  |  |  |  |
| Kind-supportive | 4.68 | 0.97 | 5.03 | 0.78 | 385 | −5.70 | <.001 | −0.40 | [−0.54, −0.25] |
| - Kind | 4.61 | 1.18 | 5.05 | 0.99 | 381 | −5.76 | <.001 | −0.41 | [−0.55, −0.26] |
| - Supportive | 4.75 | 1.07 | 5.02 | 0.89 | 384 | −3.79 | <.001 | −0.27 | [−0.41, −0.13] |
| Financially secure-successful | 3.39 | 1.16 | 3.90 | 1.06 | 485 | −6.45 | <.001 | −0.45 | [−0.60, −0.31] |
| - Financially secure | 3.09 | 1.45 | 4.31 | 1.38 | 384 | −3.89 | <.001 | −0.28 | [−0.42, −0.14] |
| - Successful ambitious | 3.69 | 1.36 | 4.66 | 1.23 | 384 | −7.04 | <.001 | −0.48 | [−0.63, −0.34] |
|  |  |  |  |  |  |  |  |  |  |
| Educated-intelligent | 4.46 | 0.89 | 4.62 | 0.91 | 385 | −2.54 | .01 | −0.17 | [−0.31, −0.04] |
| - Educated | 4.48 | 0.98 | 4.66 | 1.03 | 384 | −2.50 | .01 | −0.18 | [−0.32, −0.04] |
| - Intelligent | 4.44 | 1.02 | 4.58 | 1.00 | 384 | −1.96 | .05 | −0.13 | [−0.27, 0.00] |

*Note.* Scale ranged from 0–6.

## S14 Results for Additional Analyses Controlling for Influence of Relationship Interests on Partner Preferences

**Table S14**

*Summary of Analyses Controlling for Relationship Interests*

| Ideal partner preferences | Asexual  Women | | Heterosexual Women | | *t* | *df* | *p* | *d* | 95% CI |
| --- | --- | --- | --- | --- | --- | --- | --- | --- | --- |
|  | *M* | *SD* | *M* | *SD* |  |  |  |  |  |
| 1) Controlling for disinterest in long-term relationships (*N* = 636) |  |  |  |  |  | 317 |  |  |  |
| Confident-assertive | 3.91 | 1.16 | 4.40 | 1.00 | −6.05 |  | <.001 | **−0.45** | [−0.61, −0.30] |
| Attractive | 3.18 | 1.52 | 3.86 | 1.27 | −6.14 |  | <.001 | **−0.49** | [−0.66, −0.32] |
| Sexually experienced | 1.32 | 1.81 | 2.50 | 2.02 | −8.35 |  | <.001 | **−0.61** | [−0.77, −0.46] |
| Kind-supportive | 5.47 | 0.66 | 5.56 | 0.57 | −1.73 |  | .09 | −0.14 | [−0.30, 0.02] |
| Financially secure-successful | 4.09 | 1.21 | 4.65 | 1.05 | −6.36 |  | <.001 | **−0.50** | [−0.66, −0.33] |
| Educated-intelligent | 4.90 | 1.04 | 4.87 | 1.12 | 0.39 |  | .70 | 0.03 | [−0.12, 0.19] |
| 2) + interest sexual, non-romantic relationships (*N* = 636) |  |  |  |  |  | 317 |  |  |  |
| Confident-assertive | 3.91 | 1.16 | 4.43 | 0.97 | −6.23 |  | <.001 | **−0.49** | [−0.65, −0.33] |
| Attractive | 3.18 | 1.52 | 3.79 | 1.32 | −5.40 |  | <.001 | **−0.43** | [−0.60, −0.27] |
| Sexually experienced | 1.32 | 1.81 | 2.02 | 1.98 | −4.93 |  | <.001 | −0.37 | [−0.52, −0.22] |
| Kind-supportive | 5.47 | 0.66 | 5.61 | 0.53 | −3.07 |  | .00 | −0.23 | [−0.38, −0.08] |
| Financially secure-successful | 4.09 | 1.21 | 4.60 | 1.09 | −5.72 |  | <.001 | **−0.44** | [−0.60, −0.28] |
| Educated-intelligent | 4.90 | 1.04 | 4.95 | 1.00 | −0.57 |  | .57 | −0.04 | [−0.19, 0.11] |
| 3) + parenting desire (*N* = 634) |  |  |  |  |  | 316 |  |  |  |
| Confident-assertive | 3.91 | 1.16 | 4.29 | 0.99 | −4.59 |  | <.001 | −0.35 | [−0.51, −0.20] |
| Attractive | 3.18 | 1.52 | 3.79 | 1.30 | −5.53 |  | <.001 | **−0.43** | [−0.59, −0.27] |
| Sexually experienced | 1.31 | 1.79 | 2.46 | 2.08 | −7.59 |  | <.001 | **−0.59** | [−0.76, −0.43] |
| Kind-supportive | 5.47 | 0.66 | 5.51 | 0.61 | −0.77 |  | .44 | −0.06 | [−0.22, 0.10] |
| Financially secure-successful | 4.09 | 1.21 | 4.48 | 1.13 | −4.16 |  | <.001 | −0.33 | [−0.49, −0.17] |
| Educated-intelligent | 4.90 | 1.04 | 4.95 | 1.12 | −0.64 |  | .53 | 0.05 | [−0.11, 0.20] |

*Note.* Sample sizes are given in brackets. Substantial effect estimates are in bold. Scale ranged from 0–6.

## S15 Effect Estimates and 95% Confidence Intervals for Additional Analyses Controlling for Relationship Interests

**Figure S15**

*Effect Sizes and 95% Confidence Intervals for Analyses Controlling for Relationship Interests*


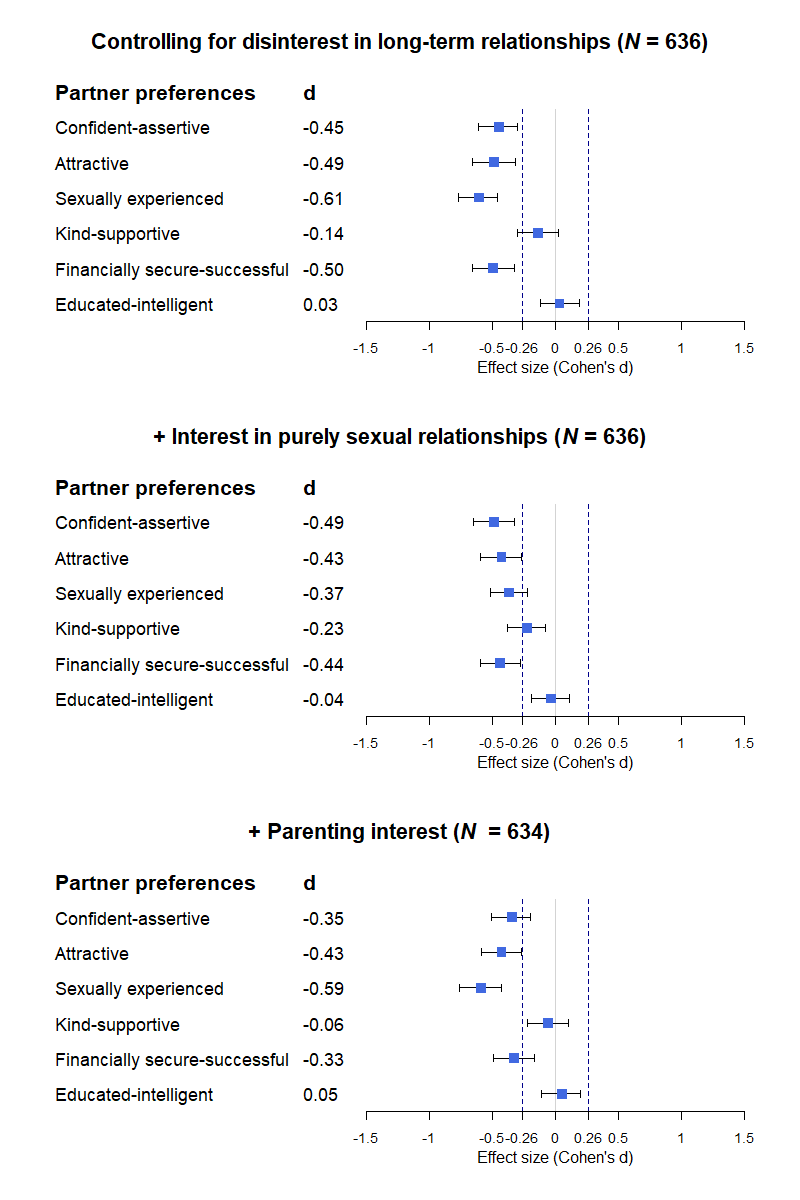


*Note.* Effect sizes (Cohen’s *d*s) with their 95% confidence interval are displayed. Vertical dotted lines indicate the SESOI boundaries (*d* = (−)0.26).

## S16 Comparison of Balance Properties Before and After Matching for the Robustness Check

**Table S16**

*Balance Properties Before Matching and After Matching for all Robustness Analysis Samples*

| Covariates | Before matching  (*N* = 48,655) | | Relationship options sample  (*N* = 758) | | Partner preference sample  (*N* = 920) | | Self-rating sample  (*N* = 902) | |
| --- | --- | --- | --- | --- | --- | --- | --- | --- |
|  | *SMD* | *VR* | *SMD* | *VR* | *SMD* | *VR* | *SMD* | *VR* |
| Propensity score | 0.46 | 176.31 | 0.21 | 3.00 | 0.23 | 3.46 | 0.21 | 3.34 |
| Gender |  |  |  |  |  |  |  |  |
| - Woman | −0.34 | - | −0.19 | - | −0.18 | - | −0.17 | - |
| - Man | −0.07 | - | 0.00 | − | 0.00 | - | 0.02 | - |
| - Genderqueer/Non-binary | 0.39 | - | 0.20 | - | 0.19 | - | 0.18 | - |
| Language |  |  |  |  |  |  |  |  |
| - English | 0.56 | - | 0.02 | - | 0.04 | - | 0.05 | - |
| - German | 0.03 | - | 0.01 | - | −0.02 | - | −0.02 | - |
| - Danish | −0.10 | - | −0.02 | - | 0.02 | - | −0.03 | - |
| - French | −0.49 | - | −0.07 | - | −0.10 | - | −0.06 | - |
| - Japanese | 0.06 | - | 0.03 | - | 0.02 | - | 0.01 | - |
| - Chinese | −0.08 | - | 0.00 | - | 0.00 | - | 0.05 | - |
| - Portuguese | −0.12 | - | 0.01 | - | 0.02 | - | 0.01 | - |
| - Spanish | −0.35 | - | −0.02 | - | −0.01 | - | −0.05 | - |
| - Russian | 0.02 | - | 0.06 | - | 0.05 | - | 0.06 | - |
| - Italian | −0.15 | - | 0.00 | - | −0.01 | - | 0.00 | - |
| Age | −0.26 | 0.65 | 0.00 | 0.93 | −0.01 | 0.89 | −0.01 | 0.98 |
| Relationship status |  |  |  |  |  |  |  |  |
| - Long-term relationship | −0.80 | - | 0.06 | - | 0.05 | - | 0.04 | - |
| - Dating/Sexual relationship | −0.33 | - | 0.00 | - | −0.02 | - | −0.01 | - |
| - No relationship | 0.92 | - | −0.06 | - | −0.04 | **-** | −0.03 | - |
| Relationship length | −0.38 | 0.52 | 0.04 | 0.99 | 0.04 | 1.16 | 0.03 | 1.14 |
| Discarded^a^ | - | | 0 | | 0 | | 0 | |
| Unbalanced^b^ | - | | 8 | | 7 | | 5 | |

*Note. SMD* = Standardized mean difference. *VR* = Variance ratio. Sample sizes are given in brackets. Country is not included in the table because of space restrictions. Nearest-neighbor-matching algorithms were run without replacement.

^a^ Discarded refers to number of asexual participants that could not be matched during the matching process.

^b^ Unbalanced refers to the number of covariates that could not be balanced through matching (including the country variables).

## S17 Love Plot Displaying Standardized Mean Differences Before and After Matching of all Covariates for the Robustness Check

**Figure S17**

*Standardized Mean Differences Across all Covariates Before and After Matching for the Robustness Analysis of the Partner Preferences Sample*

*
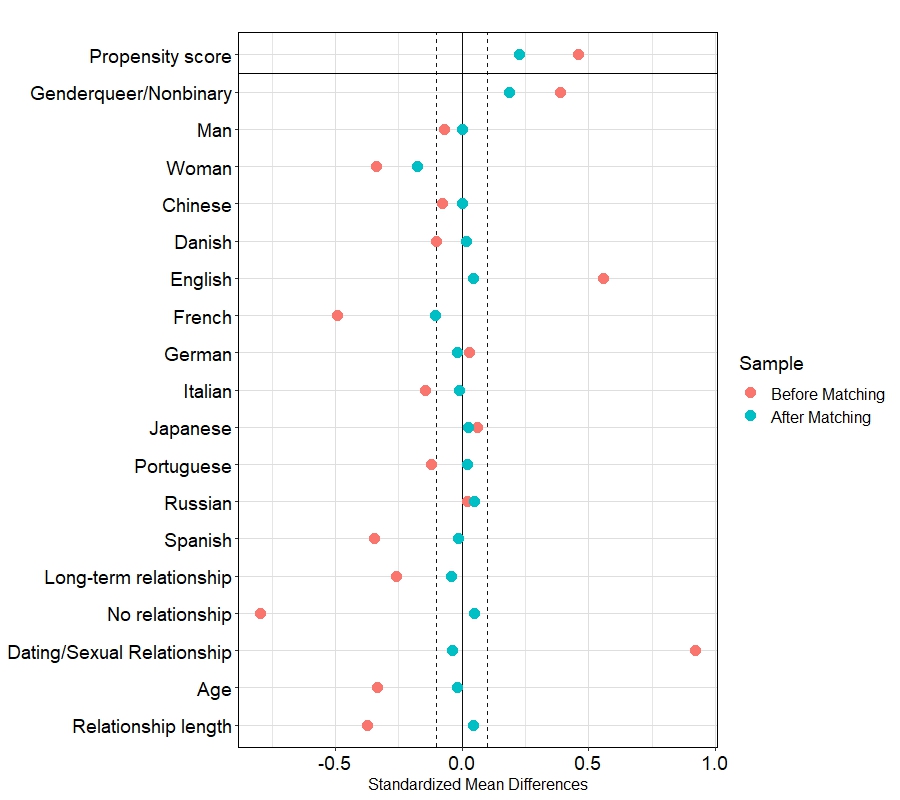
*

*Note.* The dotted vertical lines indicate the cut off of (−)0.1 standardized mean difference (SMD). Red dots indicate SMDs before matching. Blue dots indicate SMDs after matching. SMDs > 0 indicate that heterosexual individuals have higher mean scores. SMDs < 0 indicate that asexual individuals have higher mean scores. The country variables were omitted from the figure due to space restrictions and a clearer visualization. Nearest-neighbor-matching without caliper adjustment was run. Values are based on the partner preference sample (*N* = 920).

## S18 Distributions of Propensity Scores in the Matched and Unmatched Asexual and Heterosexual Samples for the Robustness Analysis

**Figure S18**

*Distributions of Propensity Scores in the Matched and Unmatched Asexual and Heterosexual Samples for the Robustness Analysis*

*
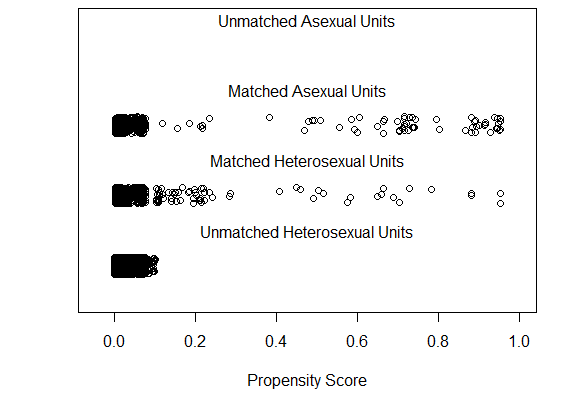
*

*Note.* Represented are the distributions for the partner preference analysis sample. There are no unmatched asexual units displayed because no asexual participant was discarded during the matching procedure.

## S19 Results of t-Tests for the Robustness Check

**Table S19**

*Summary of Robustness Analysis*

| Outcome | Asexual Individuals | | Heterosexual Individuals | | *t* | *df* | *p* | *d* | 95% CI |
| --- | --- | --- | --- | --- | --- | --- | --- | --- | --- |
|  | *M* | *SD* | *M* | *SD* |  |  |  |  |  |
| Preferred relationship options (*N* = 758) |  |  |  |  |  | 378 |  |  |  |
| Sexual, non-romantic relationship(s) | 0.72 | 1.32 | 1.69 | 1.83 | −8.42 |  | <.001 | **−0.61** | [−0.76, −0.45] |
| Non-sexual, romantic relationship(s) | 4.41 | 1.71 | 2.33 | 1.99 | 16.01 |  | <.001 | **1.12** | [0.94, 1.29] |
| Monogamous relationship(s) | 4.16 | 1.77 | 4.95 | 1.59 | −6.75 |  | <.001 | **−0.46** | [−0.61, −0.32] |
| Non-monogamous relationship(s) | 1.37 | 1.77 | 0.89 | 1.49 | 4.25 |  | <.001 | 0.29 | [0.15, 0.43] |
| Alternative committed relationship(s) | 4.58 | 1.64 | 3.41 | 2.15 | 8.69 |  | <.001 | **0.61** | [0.46, 0.76] |
| Being non-partnered | 3.29 | 1.88 | 2.11 | 1.69 | 9.68 |  | <.001 | **0.66** | [0.51, 0.80] |
| Becoming a parent | 2.32 | 2.16 | 3.87 | 2.16 | −10.21 |  | <.001 | **−0.72** | [−0.87, −0.56] |
|  |  |  |  |  |  |  |  |  |  |
| Ideal partner preferences (*N* = 920) |  |  |  |  |  | 459 |  |  |  |
| Confident-assertive | 3.89 | 1.15 | 4.34 | 0.97 | −6.92 |  | <.001 | **−0.43** | [−0.55, −0.30] |
| Attractive | 3.03 | 1.55 | 3.90 | 1.17 | −9.65 |  | <.001 | **−0.63** | [−0.77, −0.49] |
| Sexually experienced | 1.28 | 1.80 | 2.60 | 1.93 | −11.15 |  | <.001 | **−0.70** | [−0.84, −0.57] |
| Kind-supportive | 5.45 | 0.72 | 5.52 | 0.58 | −1.54 |  | .13 | −0.10 | [−0.23, 0.03] |
| Financially secure- successful | 4.04 | 1.24 | 4.32 | 1.12 | −3.62 |  | <.001 | −0.24 | [−0.37, −0.11] |
| Educated-intelligent | 4.84 | 1.07 | 4.90 | 1.02 | −0.75 |  | .45 | −0.05 | [−0.18, 0.08] |
|  |  |  |  |  |  |  |  |  |  |
| Self-ratings (*N* = 902) |  |  |  |  |  | 450 |  |  |  |
| Confident-assertive | 3.18 | 1.31 | 3.74 | 1.14 | −7.23 |  | <.001 | **−0.46** | [−0.59, −0.33] |
| Attractive | 3.04 | 1.28 | 3.62 | 1.10 | −7.57 |  | <.001 | **−0.49** | [−0.62, −0.35] |
| Sexually experienced | 1.08 | 1.46 | 2.16 | 1.82 | −11.17 |  | <.001 | **−0.65** | [−0.78, −0.53] |
| Kind-supportive | 4.66 | 0.96 | 5.00 | 0.80 | −5.88 |  | <.001 | −0.39 | [−0.52, −0.25] |
| Financially secure-successful | 3.34 | 1.18 | 3.85 | 1.09 | −6.96 |  | <.001 | **−0.45** | [−0.58, −0.32] |
| Educated-intelligent | 4.42 | 0.92 | 4.59 | 0.89 | −2.89 |  | .00 | −0.18 | [−0.31, −0.06] |

*Note.* Sample sizes are given in brackets. Substantial effect estimates are in bold. Scale ranged from 0*–*6.

## S20 Effect Sizes and 95% Confidence Intervals for the Robustness Analyses

**Figure S20**

*Effect Sizes and 95% Confidence Intervals for the Robustness Analyses*

*
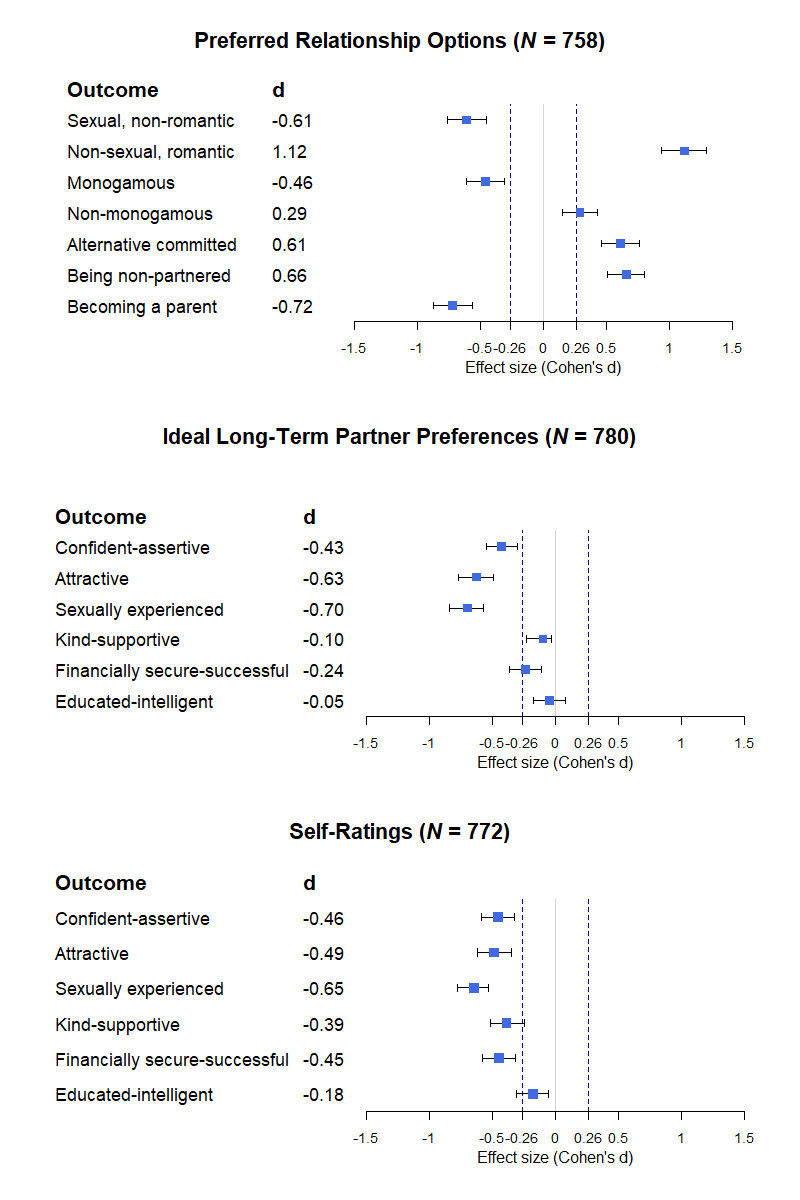
*

*Note.* Effect sizes (Cohen’s *d*s) with their 95% confidence interval are displayed. Vertical dotted lines indicate the SESOI boundaries (*d* = (−)0.26).

## S21 Additional Sensitivity Analysis Using E-values

In addition to Rosenbaum’s sensitivity analysis, the E-value (VanderWeele & Ding, 2017) was calculated. The E-value describes how large the association of an unobserved confounder with treatment and outcome would need to be to explain away an observed effect. A large E-value implies that unmeasured confounding would need to be relatively substantial to explain away an effect. Conversely, a small E-value implies that unmeasured confounding only would need to be little to be able to explain away an effect. E-values were not only estimated for the effect estimate, but also for the estimate’s confidence interval limit that is closer to null. Additionally, so-called “non-null E-values” were calculated. Non-null E-values describe the strength of association that an unmeasured confounder would need to have with both the outcome and the predictor to shift the effect estimate to a specified effect size that would be considered practically irrelevant (rather than null). For the purpose of the present study, this value was defined as the SESOI. Again, E-values were calculated for each estimate as well as its confidence interval limit closer to null. The E-value is measured on the risk ratio scale. Cohen’s *d* was therefore transformed into risk ratio values. Importantly, the E-value allows for comparisons across studies as it is a standardized metric. E-values were calculated using the package EValue v4.1.3 (Mathur et al., 2018).

The following section focusses on non-null E-values for the effect estimate. E-values for preferred relationship outcomes ranged from 1.27 (non-monogamous relationships) to 3.84 (non-sexual, romantic relationships). To explain away the substantiality of an observed estimate of *d* = −0.46 for interest in monogamous relationships, an unmeasured confounder would need to be associated with both the outcome (i.e., interest in monogamous relationships) and the predictor (i.e., sexual orientation) by a risk ratio of RR = 1.72–fold each, above and beyond the measured confounders. A weaker confounding could not explain it away (VanderWeele & Ding, 2017).

With an observed estimate of *d* = 1.12 for interest in non-sexual, romantic relationships, an unmeasured confounder that was associated with both the outcome (i.e., interest in non-sexual, romantic relationships) and the predictor (i.e., sexual orientation) by a risk ratio of RR = 3.84–fold each, above and beyond the measured confounders, could explain away the substantiality of the estimate, but a weaker confounding could not. Thus, the evidence for the effect estimate from the E-value seems reasonably strong.

For partner preference outcomes E-values ranged from 1.88 (attractive) to 2.26 (sexually experienced). This means that with an observed estimate of *d* = −0.52 for the preference for an attractive partner, unmeasured confounding would need to have an association with both the outcome and sexual orientation by a risk ratio of RR = 1.88–fold each, above and beyond the measured confounders, to explain away the substantiality of the estimate. Similarly, with an observed estimate of *d* = −0.66 for the preference for a sexually experienced partner, unmeasured confounding would need to have an association with both the outcome and the sexual orientation by a risk ratio of RR = 2.26–fold each, above and beyond the measured confounders, to explain away the substantiality of the estimate.

The E-values for the self-rating outcomes ranged from 1.64 (confident-assertive) to 2.23 (sexually experienced). Again, with an observed estimate of *d* = −0.43 for confident-assertive, unmeasured confounding needs to be associated with both outcome and sexual orientation by a risk ratio of RR = 1.64 to explain away the substantiality of the estimate. And with an observed estimate of *d* = −0.65 for sexually experienced, unmeasured confounding needs to be associated with both outcome and sexual orientation by a risk ratio of RR = 2.23 to explain away the substantiality of the estimate.

**Table S17**

*E-Values for all Statistically Significant Results*

| Outcome | E-Value | | | |
| --- | --- | --- | --- | --- |
|  | For *d* | For CI | Non-null | |
|  |  |  | For *d* | For CI |
| Preferred relationship options (*N* = 646) |  |  |  |  |
| Sexual, non-romantic relationship(s) | 3.08 | 2.56 | 2.29 | 1.84 |
| Non-sexual, romantic relationship(s) | 4.99 | 4.07 | 3.84 | 3.10 |
| Monogamous relationship(s) | 2.41 | 1.97 | 1.72 | 1.28 |
| Non-monogamous relationship(s) | 1.69 | 1.26 | 1.27 | 1.00 |
| Alternative committed relationship(s) | 2.75 | 2.27 | 2.04 | 1.61 |
| Being non-partnered | 2.88 | 2.39 | 2.09 | 1.67 |
| Becoming a parent | 3.45 | 2.85 | 2.56 | 2.07 |
|  |  |  |  |  |
| Ideal partner preferences (*N*= 780) |  |  |  |  |
| Confident-assertive | 2.15 | 1.80 | 1.47 | 1.00 |
| Attractive | 2.59 | 2.16 | 1.88 | 1.48 |
| Sexually experienced | 3.05 | 2.57 | 2.26 | 1.86 |
| Kind-supportive | 1.58 | 1.16 | 1.42 | 1.00 |
| Financially secure/successful | 1.72 | 1.33 | 1.27 | 1.00 |
|  |  |  |  |  |
| Self-ratings (*N* = 772) |  |  |  |  |
| Confident-assertive | 2.32 | 1.93 | 1.64 | 1.23 |
| Attractive | 2.50 | 2.08 | 1.80 | 1.41 |
| Sexually experienced | 3.01 | 2.59 | 2.23 | 1.87 |
| Kind-supportive | 2.23 | 1.85 | 1.56 | 1.10 |
| Financially secure-successful | 2.38 | 1.97 | 1.69 | 1.28 |
| Educated-intelligent | 1.61 | 1.21 | 1.36 | 1.00 |

*Note. d* = Cohen’s d. CI = confidence interval*.* Sample sizes are given in brackets. E-values are displayed for the effect estimate (“For *d*”) as well as for its 95% confidence interval limit that is closer to null (“For *CI*”). Additionally, non-null E-Values are displayed for the effect estimate and its confidence interval limit closer to null. More specifically, the non-null value was set at *d* = (−).26 which represents the SESOI boundaries.

**References**

Mathur, M. B., Ding, P., Riddell, C. A., & VanderWeele, T. J. (2018). Web site and R Package for computing E-values. *Epidemiology*, *29*(5), e45–e47. <https://doi.org/10.1097/EDE.0000000000000864>

VanderWeele, T. J., & Ding, P. (2017). Sensitivity analysis in observational research: Introducing the E-value. *Annals of Internal Medicine*, *167*(4), 268–274. <https://doi.org/10.7326/M16-2607>
